# Neratinib enhances the efficacy of CDK4/6 inhibitor plus endocrine therapy in HR^+^/HER2-low breast cancer cell line ZR-75-1 via hsa-miR-23a-5p

**DOI:** 10.1038/s41598-024-82137-9

**Published:** 2024-12-28

**Authors:** Liushan Chen, Lingling Ye, Yuqi Liang, Wei Luo, Qian Zuo, Ping Huang, Yuyu Hu, Yan Dai, Yingchao Wu, Qianqian Guo, Qianjun Chen

**Affiliations:** 1 Chinese Medicine Guangdong Laboratory, Hengqin, 519031 Guangdong China; 2 Breast Disease Clinical Transformation Team, The Second Affiliated Hospital of Guangzhou, University of Chinese Medicine, Guangzhou, 510120 Guangdong China; 3 Breast Department, Guangdong Provincial Hospital of Chinese Medicine, Guangzhou, 510120 Guangdong China; 4 The Second Clinical College of Guangzhou University of Chinese Medicine, Guangzhou, 510006 Guangdong China; 5 Chinese Medicine Guangdong Laboratory (Hengqin Laboratory), Zhuhai, 519031 Guangdong China

**Keywords:** Neratinib, HER2 mRNA stability, HR^+^/HER2-low, CDK4/6 inhibitor, Endocrine therapy, Hsa-miR-23a-5p, Breast cancer, Cancer

## Abstract

HR^+^/HER2-low breast cancer is a significant subgroup of conventional HR^+^/HER2-negative breast cancer, and combination of CDK4/6 inhibitor and endocrine therapy is the standard first-line and second-line treatments for advanced HR^+^/HER2-low breast cancer. Nevertheless, it remains uncertain whether HER2 signaling affects the effectiveness of CDK4/6 inhibitor administered in combination with endocrine therapy for HR^+^/HER2-low breast cancer and suitable intervention measures. This study revealed poor efficacy for CDK4/6 inhibitor combined with endocrine therapy for HR^+^/HER2-low breast cancer in vitro and in vivo models. Secondly, suppression of HER2 gene expression in HR^+^/HER2-low breast cancer cells resulted in significantly improved efficacy for CDK4/6 inhibitor combined with endocrine therapy. Furthermore, the anti-HER inhibitor neratinib was administered to enhance the effectiveness of CDK4/6 inhibitor combined with endocrine therapy in HR^+^/HER2-low breast cancer by inhibiting the HER2 pathway and lowering HER2 mRNA expression. Strikingly, neratinib reversed the efficacy of CDK4/6 inhibitor and endocrine therapy by reducing HER2 mRNA stability in HR^+^/HER2-low breast cancer through the interaction of HER2 3’-UTR region with hsa-miR-23a-5p. Even after reducing neratinib dosage to the standard 1/2 dose (20 mg/kg), it remained highly effective and well-tolerated. This study provides a viable and well-tolerated triple combination therapy for clinical HR^+^/HER2-low breast cancer.

**Supplementary Information:**

The online version contains supplementary material available at 10.1038/s41598-024-82137-9.

## Introduction

Advanced hormone receptor (HR)-positive(+)/Human epidermal growth factor receptor 2 (HER2)-negative(-) breast cancer represents an important clinical challenge, for which the combination of cyclin-dependent kinases 4 and 6 (CDK4/6) inhibitors with endocrine therapy has emerged as the standard conventional option for both first-line and second-line treatments^1–6^. This therapeutic approach is also used as an adjuvant therapy for early-stage HR^+^/HER2^−^ subtypes in high-risk populations^7–9^. HER2^−^ subtypes include HER2 IHC 0 as HER2-0 and IHC score 1^+^ and 2^+^/ISH non-amplified as HER2-low^10–13^. Among the HR^+^/HER2^−^ subset, HR^+^/HER2-low patients constitute a significant proportion, accounting for 60.9–77.3% of cases^14,15^.

The effectiveness of the combination of CDK4/6 inhibitors with endocrine therapy in individuals with HR^+^/HER2-0 and HR^+^/HER2-low phenotypes remains unknown. Clinical findings suggest that HR^+^/HER2-low cases have decreased survival benefit from CDK4/6 inhibitors combined with endocrine therapy compared with HR^+^/HER2-0 counterparts^16–18^. This implies a potential effect of HER2-low status on the response to CDK4/6 inhibitors in HR^+^/HER2- breast cancer, possibly associated with resistance mechanisms. Nevertheless, another study reported contradictory results, demonstrating enhanced efficacy for aromatase inhibitors in combination with CDK4/6 inhibitors in the HR^+^/HER2-low subgroup^19^. Subsequent retrospective analyses also proposed that CDK4/6 inhibitors combined with endocrine therapy may not show reduced efficacy in all HR^+^/HER2-low breast cancer cases, and it is likely that HER2 expression may be a contributing factor^20–22^. Despite these insights, experimental validation and elucidation of the underpinning biological mechanisms have not been performed, and interventions governing the impact of HER2-low expression on the response to CDK4/6 inhibitors combined with endocrine therapy in HR^+^/HER2-low breast cancer remain elusive.

While molecular targeted agents such as trastuzumab and pertuzumab have shown limited efficacy in HER2-low breast cancer subtype^23,24^, small molecule tyrosine kinase inhibitors (TKIs) are promising in individuals with HER2 mutations within the advanced HR^+^/HER2^−^ subgroup^25^. Importantly, in vitro assays have highlighted the ability of neratinib to effectively reduce total HER2 levels in HER2-low breast cancer cells, resulting in profound growth inhibition^26–29^. This finding raises the intriguing question of whether neratinib potentially reverses resistance to CDK4/6 inhibitors combined with endocrine therapy, because of low HER2 expression in HR^+^/HER2-low breast cancer. However, existing evidence supporting this hypothesis is limited.

The present study aimed to evaluate the suppressive effect of CDK4/6 inhibitor in combination with endocrine therapy in HR^+^/HER2-low breast cancer and to explore the specific mechanism by which neratinib improves the efficacy of CDK4/6 inhibitor in combination with endocrine therapy. This was achieved by using HR^+^/HER2-low cell lines with low HER2 expression. Additionally, this work investigated whether neratinib could reverse this effect, thereby exploring enhanced therapeutic options involving CDK4/6 inhibitors for HR^+^/HER2-low breast cancer.

## Materials and methods

### Cell lines and cell culture

HR^+^/HER2^+^ breast cancer BT474, HR^+^/HER2-low breast cancer ZR-75-1, and HR^+^/HER2-0 breast cancer T47D cell lines were obtained from the American Type Culture Collection (ATCC, Manassas, VA, USA). BT474 and T47D cells were cultured in DMEM supplemented with 10% fetal bovine serum (Sigma-Aldrich, Germany) and 100 units/ml penicillin and streptomycin (Gibco, USA). T47D cells were treated with 100 nM fulvestrant (MedChem Express, USA) and 150 nM palbociclib (MedChem Express, USA). ZR-75-1 cells were cultured in RPMI 1640 supplemented with 10% fetal bovine serum and 100 units/ml penicillin and streptomycin. ZR-75-1 cells were administered 100 nM fulvestrant, 100–1600 nM palbociclib, and 125–2000 nM neratinib (MedChem Express, USA).

### Immunofluorescence

Adherent cells were washed with PBS, fixed with 4% paraformaldehyde, permeabilized with 0.1% Triton X-100, and blocked with 1% bovine serum albumin (BSA) in PBS. Cells were incubated with anti-HER2 antibody (1:300; 18299-1-AP, Proteintech, China) in 1% BSA at 4 °C overnight, washed three times with PBS, and then incubated with Goat Anti-Rabbit IgG H&L (Alexa Fluor^®^ 488) (Abcam, England, ab150077, 1:1000). DNA staining was performed with Gold Antifade Mountant with DAPI (Invitrogen, USA, P36931). Immunofluorescence data were analyzed by Leica SP5 confocal laser scanning microscopy (Leica Microsystems, Buffalo Grove, USA).

### Cell viability and combination effect analyses

Cells were seeded in 96-well plates at 3000/well for 12 h and treated with 100 nM fulvestrant and different concentrations of palbociclib (0, 100, 200, 400, 800, and 1600 nM) in combination, as well as with the triple combination of fulvestrant, palbociclib, and neratinib (0, 125, 250, 500, 1000, and 2000 nM), for 48 h. Cell viability was assessed by the Cell Counting Kit-8 (CCK-8) assay. Optical density (OD) was measured at 450 nm on a microplate reader (Bio-Rad Model550, CA) after a 2-hour incubation. The combination index (CI) was calculated with the CompuSyn software. CI values of 0.9–1.1, 0.3–0.9 and < 0.3 were considered to indicate additive, synergistic, strongly synergistic effects; values > 1.1 indicated antagonism. The median inhibitory concentration (IC50) was determined with Prism 8 (GraphPad software).

### Colony formation assay

Eight hundred cells were seeded in six-well plates for 24 h at 37 °C, cultured with a medium containing 10% FBS and treated with 100 nM fulvestrant and 200 nM palbociclib in combination, as well as the triple combination of fulvestrant, palbociclib, and 125 nM neratinib, for 15 days. After fixation with 4% paraformaldehyde for 20 min, the plates were stained with crystal violet (Beyotime, Jiangsu, China) for 15 min. The colonies were counted with the ImageJ software (ImageJ 1.53e, Maryland, USA) after PBS washes and air-drying.

### RNA stability assay

To confirm RNA stability, the RNA decay test was performed with actinomycin D (ActD) (MCE, USA, HY-1559). At 15 µg ActD, breast cancer cells were harvested from 6-well plates at 0, 5 and 10 h for qRT-PCR. The relative mRNA expression levels at 0 h were standardized to 1.

### MicroRNA sequencing

Using the TRIzol reagent and the miRNeasy Mini Kit, total RNA was extracted from tissues, and the TruSeq Small RNA Sample Prep kit (Illumina, San Diego, USA) was utilized to generate miRNAseq libraries. With a depth of 25 million original reads per sample, single-ended (SE) 1 × 75 base pairs were utilized for next-generation sequencing using the Illumina NextSeq 550 sequencing equipment. For miRNA quantification, the FASTQ file was sent to the Qiagen Online Data Analysis Center. We also utilized ACGT101-miR (v4.2) to analyze miRNA data.

### Reporter gene assay

Initially, TargetScan (version 7.2) was utilized to predict microRNAs that control HER2 expression. As a result, hsa-miR-23a-5p was retrieved based on the anticipated outcomes. Additionally, a double luciferase reporter gene assay kit (RG027, Beyotime) was employed for the luciferase reporter assay. The HER2-3’-UTR-wt and HER2-3’-UTR-mut luciferase reporter plasmids were synthesized by GeneChem (Shanghai, China). After inoculating ZR-75-1 cells into 96-well plates, luciferase reporter plasmids and either miR-NC or hsa-miR-23a-5p mimics were co-transfected into cells. Using the dual luciferase reporter gene assay kit (Promega, USA), relative luciferase activity was determined after 48 h of incubation. The ratio of firefly to Renilla luciferase activity was used to assess relative luciferase activity.

### Animal ethics statement

Female BALB/c-nu/nu mice (8 weeks old, 23 ± 0.5 g) were obtained from the Guangdong Laboratory Animal Center. The animals were housed in cages (six per cage) under pathogen-free conditions at 25 °C, 40–60% relative humidity, and a 12-h light/dark cycle in the experimental animal center of Guangdong Provincial Hospital of Chinese Medicine. All mice had *ad libitum* access to standard rodent chow and filtered water and were acclimatized for one week before study initiation. The animal study was approved by the Institutional Animal Ethical Committee (IAEC) of Guangdong Provincial Hospital of Chinese Medicine (Reference No. 2020031), following the Principles of Laboratory Animal Care as well as specific national laws where applicable. All experimental protocols and handling of animals followed the Guide for the Care and Use of Laboratory Animals (Institute of Laboratory Animal Research, 2011. Guide for the Care and Use of Laboratory Animals. Washington [DC]: National Academies Press).

### Breast cancer xenografts in BALB/c-nu/nu mice

After oophorectomy in mice, human T47D cells (5 × 10^6^/150 µL PBS) were injected into the mammary fat pad of each mouse. Tumor size was measured every other day with digital calipers starting on day seven after inoculation, and volume calculations were performed by multiplying the length by the width squared and divided by two. Once tumors approximately a volume of 75 mm^3^, mice were randomized into groups, with six mice per group. Two groups received the control vehicle and fulvestrant (ICI182780, 100 mg/kg in the vehicle, administered once weekly by subcutaneous injection, obtained from AstraZeneca), in combination with palbociclib (PD0332991, 150 mg/kg in the vehicle, administered five days per week orally, from Pfizer). The mammary fat pad of each mouse was injected with 5 × 10^6^ human ZR-75-1 cells in 150 µL PBS. When tumors reached approximately 75 mm^3^, mice were randomized into 3 groups of 6, including the control vehicle, fulvestrant (100 mg/kg in the vehicle, administered once weekly via subcutaneous injection) + palbociclib (150 mg/kg in the vehicle, administered five days per week by oral gavage), and triple combination (administered fulvestrant, palbociclib, and neratinib [HKI-272, 40 mg/kg in the vehicle, administered five days per week by oral gavage]) groups. Thirty days after injection of tumor cells, mice were euthanized by carbon dioxide overdose, followed by cervical dislocation. Then, tumor weights were recorded at the time of euthanasia.

### Immunohistochemistry

Tumor specimens were fixed in 4% paraformaldehyde solution for 24 h, followed by standard tissue treatment and embedding. Paraffin-embedded tumor specimens were sectioned at 4 μm and dried overnight at 37℃. The sections were dewaxed in xylene twice for 10 min each and rehydrated with a graded series of ethanol. Antigen retrieval was performed with sodium citrate buffer at 100℃ for 15 min. After incubation with 3% H_2_O_2_ for 10 min at ambient and blocking in 5% BSA in PBST for 1 h, primary antibodies were applied overnight at 4 °C. The primary antibodies employed included rabbit primary antibody ER (1:500; 21244-1-AP, Proteintech, China), and rabbit primary antibody PR (1:200; 25871-1-AP, Proteintech, China), rabbit primary antibody HER2 (1:300; 18299-1-AP, Proteintech, China). After washing, the sections were incubated with goat anti-rabbit IgG-HRP secondary antibody (1:200; Beyotime, A0208, China) for 1 h at ambient. Images were obtained with a microscopy system (Leica-DM6000B, Germany). Three randomly selected microscopic fields per slide were assessed by independent pathologists for immunohistochemical quantification.

### Plasmid constructions, cell transfection, and infection

Targeted genes were stably knocked down via lentivirus-delivered short-hairpin RNA (shRNA) delivery. PLKO.1 vectors with anti-hygromycin and anti-puromycin plasmids were constructed with appropriate primers. For shRNA knockdown and overexpression, pCDH and pLKO.1 constructs were transfected with the packaging and helper plasmids MD2G and PAX2 into 293T cells with the Calcium Phosphate Transfection Kit (CAPHOS-1KT, Sigma, Germany). Viruses were collected to infect ZR-75-1 cells along with 10 mg/mL polybrene (TR-1003-G, Sigma, Germany). Western blot was performed to assess stable cell lines for transfection efficiency.

### Western blot

Cells were cultured with fulvestrant and palbociclib combination, as well as the triple combination including fulvestrant, palbociclib, and neratinib for 48 h. Lysis was performed with the RIPA lysis buffer at 4 °C. Protein concentration was determined by the BCA protein assay. Each sample was submitted to 8% SDS-PAGE and transferred onto PVDF membranes (IPVH00010, Millipore, USA). The membranes were blocked with 5% blocking solution and incubated with primary antibodies overnight at 4 °C. The primary antibodies used in this study included rabbit antibody HER2 (1:6000; 18299-1-AP, Proteintech, China), rabbit antibody Cyclin D1 (1:1000; 2922 S, Cell Signaling Technology, USA), rabbit antibody CDK4 (1:1000; sc-23896, Santa Cruz, USA), mouse antibody EGFR (1:1000; 66455-1-PBS, Proteintech, China), and mouse antibody GAPDH (1:1000; AF0006, Beyotime, China). After washing, the membranes were incubated with goat anti-rabbit IgG-HRP secondary antibody (1:5000; A0208, Beyotime, China) for 1 h at ambient. Immunoreactive bands were then imaged on a gel image analyzer (SYSTEM GelDoc XR + IMAGELA, Bio-Rad, USA).

### Cell cycle analysis

Flow cytometry was performed to assess cell apoptosis and cell cycle distribution. Totally 2 × 10^5^ cells were equally distributed in 6-well plates. Cells were exposed to specified doses of the fulvestrant and palbociclib combination and the triple combination of fulvestrant, palbociclib, and neratinib in the exponential phase of cell growth for 24 h and subsequently digested with trypsin without EDTA. For apoptosis assessment, cells were stained with the Annexin V-FITC/PI apoptosis kit (Beyotime, Jiangsu, China). To analyze the cell cycle, cells were fixed with 70% ethanol overnight at 4℃, rinsed with PBS, and stained with the PI solution (MultiSciences, AP101-60-PI). Finally, cells were analyzed on a flow cytometer (ACEA Bioscience, NovoExpress; Agilent Technologies, Inc.) following the manufacturer’s instructions, with the NovoExpress 1.4.1 software. Three independent experiments were performed, and data were averaged.

### Statistical analysis

Data in bar graphs are mean ± standard deviation. *P* < 0.05 (versus the indicated group) was considered statistically significant and determined by one-way analysis of variance or the t-test (GraphPad Prism 8.0).

## Results

### Characterization of ZR-75-1 as an HR+/HER2-low breast cancer cell line

**Fig. 1:**
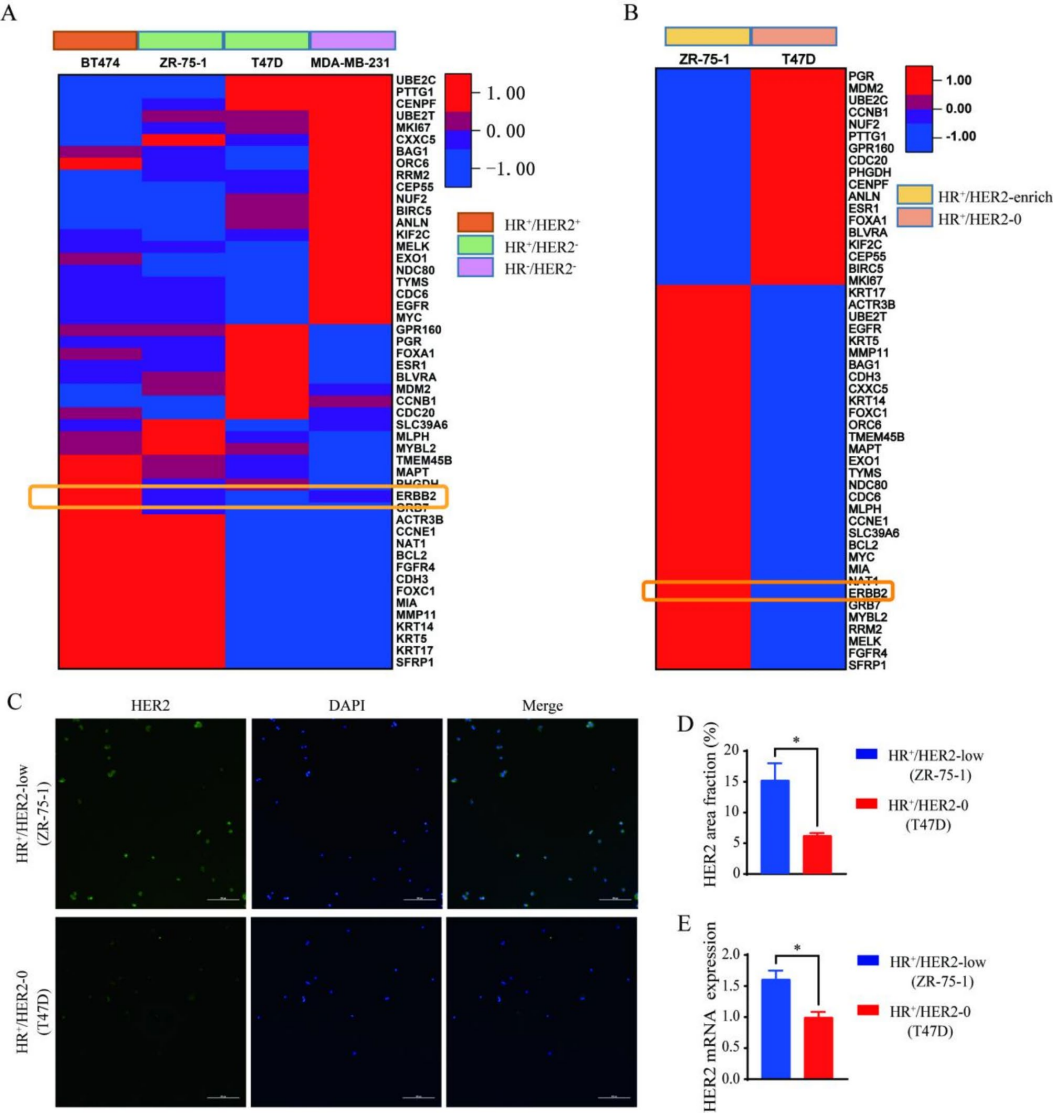
Characterization of ZR-75-1 as an HR^+^/HER2-low breast cancer cell line. (**A**,**B**) Heat maps of gene expression in PAM50 cells of different breast cancer subtypes and heat maps of ERBB2 expression in HR^+^/HER2- breast cancer cells. (**C**) HER2 expression in HR^+^/HER2-0 and HR^+^/HER2-low breast cancer subtypes by cellular immunofluorescence assay. (**D**) HER2 levels in HR^+^/HER2-0 and HR^+^/HER2-low breast cancer subtypes measured with ImageJ, *P* = 0.029. (**E**) HER2 expression in HR^+^/HER2-0 and HR^+^/HER2-low breast cancer subtypes by fluorescence quantitative PCR assay, *P* = 0.015, *P < 0.05; *n* = 3.

To assess the subtypes of HR^+^/HER2-0 and HR^+^/HER2-low breast cancer, 50 genes across diverse breast cancer cell lines were examined using PAM50 typing (GSE212143) derived from transcriptome data in the GEO database^30^. Of these, the ZR-75-1 cell line had pronounced HER2 expression compared with other breast cancer cell lines (Fig. 1A, B). Further validation by cellular immunofluorescence and fluorescence quantitative PCR assays confirmed the HR^+^/HER2-low status of ZR-75-1 cells (*P* < 0.05) (Fig. 1C-E). This compelling evidence led to the classification of ZR-75-1 as an HR^+^/HER2-low breast cancer cell line.

### The efficacy of CDK4/6 inhibitor combined with endocrine therapy in ZR-75-1 cells in vitro and in vivo

**Fig. 2:**
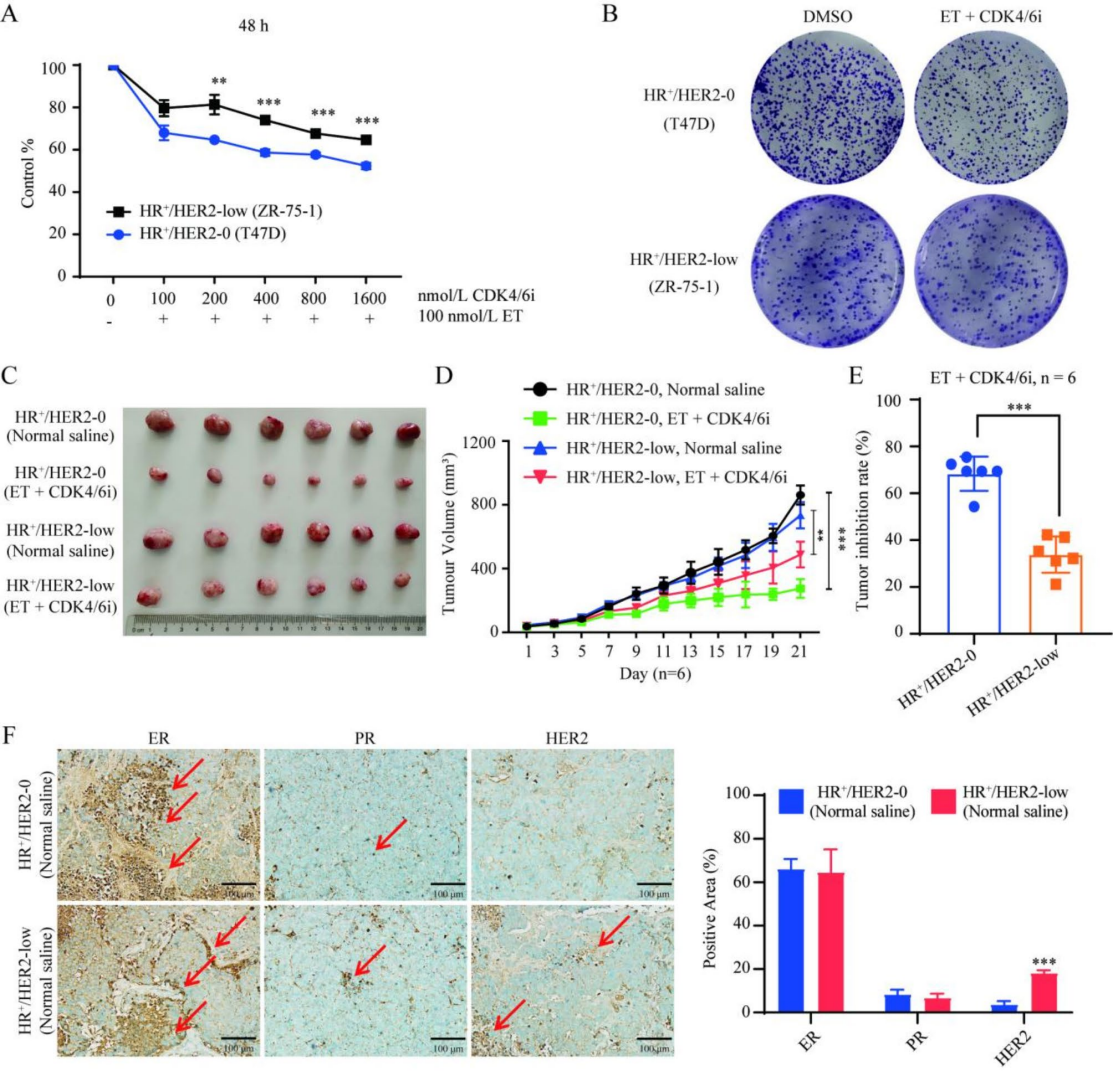
Efficacy of CDK4/6 inhibitor combined with endocrine therapy in the ZR-75-1 cell line in vitro and in vivo. (**A**,**B**) CCK-8 and colony formation assays were performed to detect the effects of various concentrations of CDK4/6 inhibitor (palbociclib) combined with endocrine therapy (100 nM, fulvestrant) on the proliferation of HR^+^/HER2-low breast cancer cells. The effect was not as significant as in HR^+^/HER2-0 breast cancer. For 200 nM CDK4/6i + 100 nM ET: HER2-low vs. HER2-0, *P =* 0.003. For 400 nM CDK4/6i + 100 nM ET: HER2-low vs. HER2-0, *P =* 0.0001. For 800 nM CDK4/6i + 100 nM ET: HER2-low vs. HER2-0, *P =* 0.0001. For 100 nM CDK4/6i + 100 nM ET: HER2-low vs. HER2-0, *P =* 0.0001. (**C**–**E**) CDK4/6 inhibitor (palbociclib, 150 mg/kg, i.g.) combined with endocrine therapy (fulvestrant, 100 mg/kg, i.m.) inhibited tumor growth in HR^+^/HER2-low breast cancer xenografted mice, but the effect was not as significant as in the HR^+^/HER2-0 subtype. For HER2-low tumor volume on day 21: normal saline vs. ET + CDK4/6i, *P =* 0.001. For HER2-0 tumor volume on day 21: normal saline vs. ET + CDK4/6i, *P =* 0.0001. For tumor inhibition rate on day 21: HER2-low vs. HER2-0, *P =* 0.0001. (**F**) ER, PR, and HER2 expression levels in tumor tissues of HR^+^/HER2-0 and HR^+^/HER2-low breast cancer xenografted mice, measured by immunohistochemistry, the arrow points to the positive area and scale bar = 100 μm. **P < 0.01; ***P < 0.001; *n* = 6. *ET* endocrine therapy, *CDK4/6i* CDK4/6 inhibitor, *ER*: estrogen receptor, *PR* progesterone receptor.

To investigate the efficacy of CDK4/6 inhibitor combined with endocrine therapy, the HR^+^/HER2-low cell line ZR-75-1 was used as the basis for cell and animal models. The outcomes were compared with those of the HR^+^/HER2-0 cell line (T47D). The collective analysis revealed that while CDK4/6 inhibitor combined with endocrine therapy effectively suppressed the proliferation of both HR^+^/HER2-0 and HR^+^/HER2-low breast cancer cells, the inhibitory effect on HR^+^/HER2-low breast cancer was notably attenuated compared with the HR^+^/HER2-0 subtype (*P* < 0.01) (Fig. 2A,B). This trend aligned with in vivo assays, in which tumor growth in HR^+^/HER2-low breast cancer xenografted mice administered CDK4/6 inhibitor combined with endocrine therapy exhibited decreased efficacy relative to the HR^+^/HER2-0 subtype (*P* < 0.05) (Fig. 2C–E). Validation of HR^+^/HER2-0 and HR^+^/HER2-low breast cancer xenografted mouse models was performed by immunohistochemistry (Fig. 2F). Collectively, these findings indicated that CDK4/6 inhibitor combined with endocrine therapy is effective in treating HR^+^/HER2-low breast cancer, but with lower efficacy compared with HR^+^/HER2-0 breast cancer.

### Role of HER2 in the poor efficacy of CDK4/6 inhibitor combined with endocrine therapy in HR^+^/HER2-low breast cancer

**Fig. 3:**
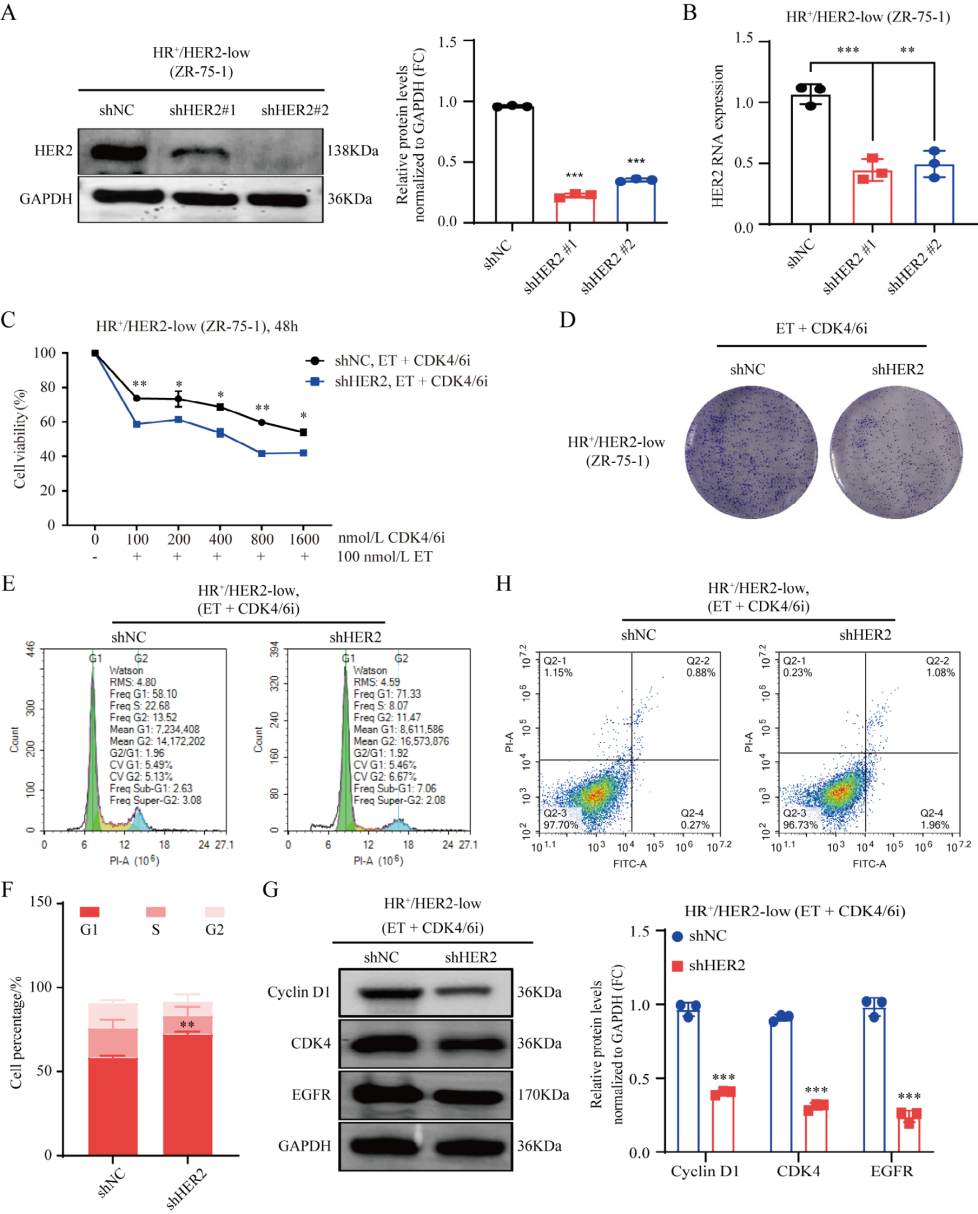
Role of HER2 in the poor efficacy of CDK4/6 inhibitor combined with endocrine therapy in HR^+^/HER2-low breast cancer. (**A**,**B**) Stable knockdown of HER2 in human HR^+^/HER2-low breast cancer cells by lentiviral shRNAs (shHER2 #1 and shHER2 #2). Knockdown effects were verified by western blot and quantitative PCR. shNC vs. shHER2 #1, *P =* 0.0009; shNC vs. shHER2 #2, *P =* 0.0019. (**C**,**D**) Knockdown of HER2 enhanced the effect of CDK4/6 inhibitor (250 nM, palbociclib) combined with endocrine therapy (100 nM, fulvestrant) in inhibiting HR^+^/HER2-low breast cancer cell proliferation as examined by the CCK-8 and colony formation experiments assays. For 100 nM CDK4/6i + 100 nM ET: shNC vs. shHER2, *P =* 0.004. For 200 nM CDK4/6i + 100 nM ET: shNC vs. shHER2, *P =* 0.045. For 400 nM CDK4/6i + 100 nM ET: shNC vs. shHER2, *P =* 0.019. For 800 nM CDK4/6i + 100 nM ET: shNC vs. shHER2, *P =* 0.001. For 1600 nM CDK4/6i + 100 nM ET: shNC vs. shHER2, *P =* 0.016. (**E**–**H**) In cell cycle assay, western blot, and apoptosis assay, knockdown of HER2 promoted G1 phase arrest and reduced Cyclin D1, CDK4 and EGFR expression. For HER2-low cells G1 phase: shNC vs. shHER2, *P =* 0.001. **P < 0.01; ***P < 0.001; *n* = 3. *ET* endocrine therapy, *CDK4/6i* CDK4/6 inhibitor.

The role of HER2 in modulating the efficacy of CDK4/6 inhibitor combined with endocrine therapy in HR^+^/HER2-low breast cancer was assessed by silencing HER2 in HR^+^/HER2-low breast cancer cells. HER2 knockdown efficiency was examined at both the RNA and protein levels (*P* < 0.001) (Fig. 3A,B). Notably, HER2 knockdown increased the inhibitory effect of CDK4/6 inhibitor combined with endocrine therapy on the proliferation of HR^+^/HER2-low breast cancer cells, as demonstrated by CCK-8 and colony formation assays (*P* < 0.05) (Fig. 3C,D). Further investigation encompassing cell cycle and apoptosis assays by flow cytometry and western blot confirmed the significant induction of G1 phase arrest, concomitant with decreased Cyclin D1, CDK4 and EGFR expression (*P* < 0.05) (Fig. 3E–H). Collectively, HER2 knockdown significantly induced G1 phase arrest, decreased the expression of Cyclin D1 and CDK4, and enhanced the inhibitory effect of CDK4/6 inhibitor combined with endocrine therapy on HR^+^/HER2-low breast cancer cells.

### Neratinib enhances the efficacy of CDK4/6 inhibitor combined with endocrine therapy in HR+/HER2-low breast cancer

**Fig. 4:**
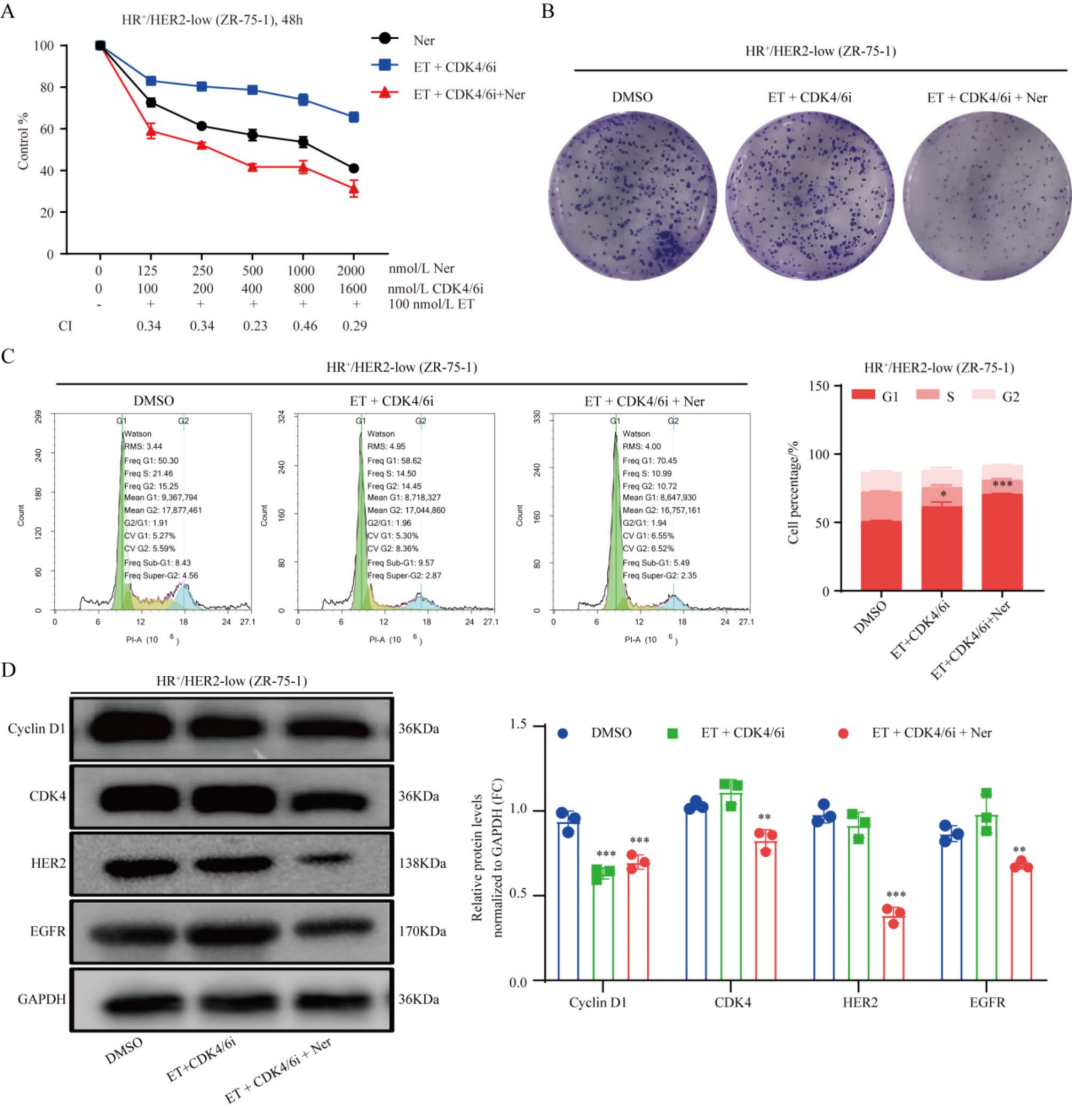
Neratinib enhances the efficacy of CDK4/6 inhibitor combined with endocrine therapy in HR^+^/HER2-low breast cancer in vitro. (**A**,**B**) CCK8 and colony formation assays were performed to assess the proliferation of HR^+^/HER2-low breast cancer cells administered various concentrations of neratinib and CDK4/6 inhibitor, respectively, combined with endocrine therapy. For HER2-low cells: ET + 100 nM CDK4/6i + 125 nM neratinib, *CI =* 0.34; ET + 200 nM CDK4/6i + 250 nM neratinib, *CI =* 0.34; ET + 400 nM CDK4/6i + 500 nM neratinib, *CI =* 0.23; ET + 800 nM CDK4/6i + 1000 nM neratinib, *CI =* 0.46; ET + 1600 nM CDK4/6i + 2000 nM neratinib, *CI =* 0.29. (**C**) The cell cycle assay was performed in HR^+^/HER2-low breast cancer cells administered the triple combination of 125 nM neratinib, CDK4/6 inhibitor (100 nM, palbociclib), and endocrine therapy (100 nM, ET, fulvestrant). For HER2-low cells’ G1 phase: DMSO vs. ET + CDK4/6i, *P =* 0.024; DMSO vs. ET + CDK4/6i + neratinib, *P =* 0.0001. (**D**) Cyclin D1, CDK4, HER2, and EGFR protein expression levels in HR^+^/HER2-0 and HR^+^/HER2-low breast cancer cells, measured by western blot. *P < 0.05; **P < 0.01; ***P < 0.001; *n* = 3. *ET*: endocrine therapy, *CDK4/6i* CDK4/6 inhibitor, *Ner* Neratinib.

This study further utilized a triple combination approach including neratinib, CDK4/6 inhibitor, and endocrine therapy, to assess the impact of HER2 on the efficacy of CDK4/6 inhibitor combined with endocrine therapy in HR^+^/HER2-low breast cancer. The outcomes clearly demonstrated that the triple combination exerted significantly enhanced inhibitory effects on proliferation and colony formation in HR^+^/HER2-low breast cancer cells, surpassing the efficacy of CDK4/6 inhibitor combined with endocrine therapy alone (CI < 0.5, *P* < 0.01) (Fig. 4A,B). Further analysis by flow cytometry and western blot unveiled the ability of neratinib to induce G1 phase arrest, accompanied by downregulation of Cyclin D1, CDK4, HER2, and EGFR (*P* < 0.05) (Fig. 4C,D). In conclusion, neratinib enhanced the efficacy of CDK4/6 inhibitor combined with endocrine therapy in HR^+^/HER2-low breast cancer.

**Fig. 5:**
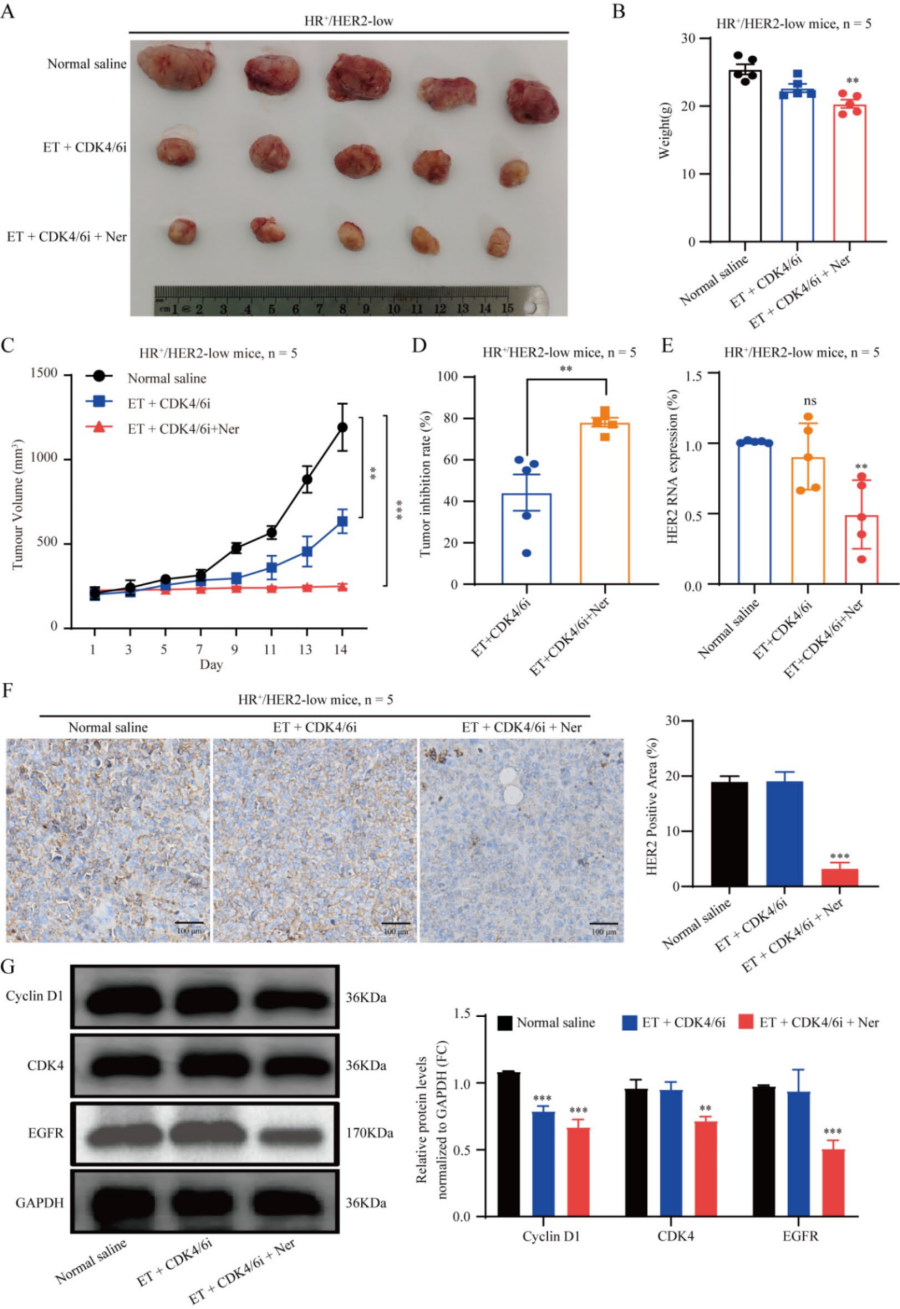
Neratinib enhances the efficacy of CDK4/6 inhibitor combined with endocrine therapy in HR^+^/HER2-low breast cancer in vivo. (**A**) Tumor photographs in animals treated with normal saline (control group), CDK4/6 inhibitor (palbociclib, 150 mg/kg, i.g.) combined with endocrine therapy (fulvestrant, 100 mg/kg, i.m.), and triple therapy consisting of standard dose of neratinib group (40 mg/kg, i.g.), CDK4/6 inhibitor, and endocrine therapy. (**B**) Weight changes in each group of HR^+^/HER2-low tumor-bearing mice. For HER2-low weight on day 14: normal saline vs. ET + CDK4/6i, *P =* 0.217; normal saline vs. standard dose ET + CDK4/6i + Ner, *P =* 0.0052. (**C**) Tumor growth curves and (**D**) tumor inhibition rates of HR^+^/HER2-low tumor-bearing mice in various groups. For HER2-low tumor volume on day 14: normal saline vs. ET + CDK4/6i, *P =* 0.009; normal saline vs. standard dose ET + CDK4/6i + Ner, *P =* 0.0003. For HER2-low tumor inhibition rate on day 14: normal saline vs. ET + CDK4/6i, *P =* 0.0055. (**E**) Tumor HER2 mRNA expression of in each group of HR^+^/HER2-low tumor-bearing mice. For HER2-low tumor HER2 mRNA expression on day 14: normal saline vs. ET + CDK4/6i, *P =* 0.381; normal saline vs. standard dose ET + CDK4/6i + Ner, *P =* 0.001. (**F**) HER2 expression in mouse HR^+^/HER2-low breast cancer tissue by IHC staining. (**G**) Cyclin D1, CDK4 and EGFR protein expression levels in HR^+^/HER2-low breast cancer, measured by western blot. *P < 0.05; **P < 0.01; ***P < 0.001; *n* = 5. *ET* endocrine therapy, *CDK4/6i* CDK4/6 inhibitor, *Ner* neratinib.

Next, to comprehensively evaluate the in vivo effect of the combination of neratinib with CDK4/6 inhibitor and endocrine therapy in HR^+^/HER2-low breast cancer, a xenograft mouse model was established with human HR^+^/HER2-low breast cancer cells. Analysis of tumor sizes in mice with the HR^+^/HER2-low breast cancer subtype revealed marked differences among the control (normal saline), CDK4/6 inhibitor + endocrine therapy, and triple combination (neratinib + CDK4/6 inhibitor + endocrine therapy groups (*P* < 0.05, *P* < 0.01). Interestingly, both the CDK4/6 inhibitor + endocrine therapy and triple combination groups displayed significant inhibition of tumor growth compared with the control group (*P* < 0.05) (Fig. 5A–D). Notably, the triple combination exhibited more pronounced effects on HER2 mRNA and protein expression levels in tumor tissues compared with CDK4/6 inhibitor combined with endocrine therapy alone (*P* < 0.01) (Fig. 5E, F). Immunoblot further verified that neratinib effectively downregulated Cyclin D1, CDK4 and EGFR by inhibiting the HER2 pathway, thus enhancing the efficacy of CDK4/6 inhibitor combined with endocrine therapy in HR^+^/HER2-low breast cancer (*P* < 0.01) (Fig. 5G).

**Fig. 6:**
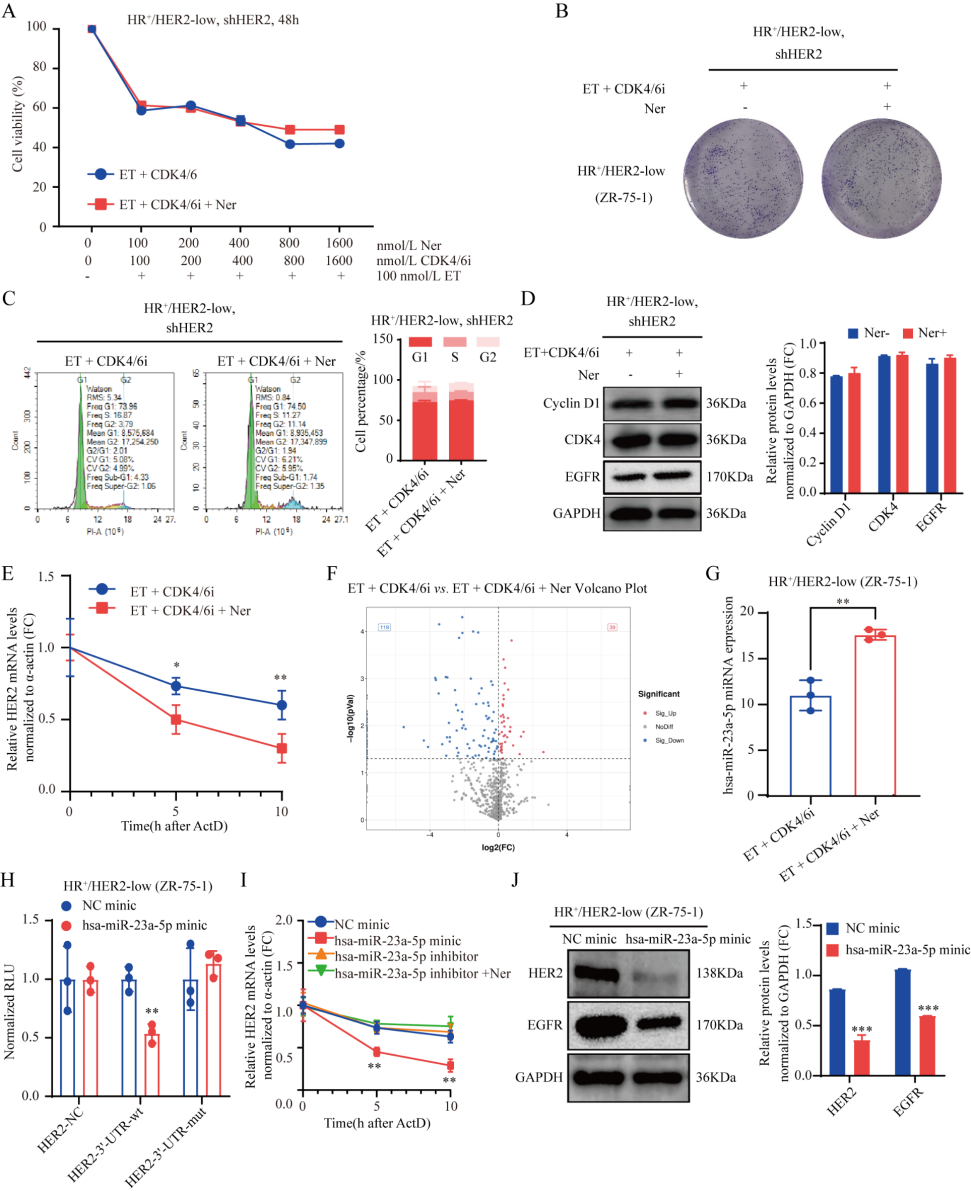
Neratinib enhances the efficacy of CDK4/6 inhibitor combined with endocrine therapy by reducing HER2 mRNA stability through the interaction of HER2’s 3’-UTR region with hsa-miR-23a-5p. (**A**,**B**) After knockdown of HER2, the effect of the triple combination of neratinib, CDK4/6 inhibitor, and endocrine therapy on HR^+^/HER2-low breast cancer cell proliferation was assessed by the CCK-8 and colony formation assays. (**C**,**D**) In cell cycle analysis and western blot, neratinib was shown to promote G1 phase arrest through the HER2 pathway, to downregulate Cyclin D1 and CDK4, and to improve the effect of CDK4/6 inhibitor combined with endocrine therapy in HR^+^/HER2-low breast cancer. (**E**) Effects of CDK4/6 inhibitor + endocrine therapy and triple combination treatment (neratinib, CDK4/6 inhibitor and endocrine therapy) on HER2 mRNA stability. MicroRNA sequencing (**F**) revealed the effect of neratinib on miRNA changes, and compared with CDK4/6 inhibitor + endocrine therapy group, the triple combination treatment group administered neratinib, CDK4/6 inhibitor, and endocrine therapy had significantly upregulated hsa-miR-23a-5p (**G**), which highly interacted with HER2’s 3’-UTR region (**H**), and significantly lowered HER2 mRNA stability (**I**) and downregulated HER2 and EGFR protein expression (**J**). *P < 0.05; **P < 0.01; ***P < 0.001; *n* = 3. *ET* endocrine therapy, *CDK4/6i* CDK4/6 inhibitor, *Ner* neratinib.

Neratinib enhanced the efficacy of CDK4/6 inhibitor combined with endocrine therapy by reducing HER2 mRNA stability through the interaction of HER2’s 3’-UTR region with hsa-miR-23a-5p. To establish the pivotal role of universal neratinib in enhancing the effectiveness of CDK4/6 inhibitor combined with endocrine therapy in HR^+^/HER2-low breast cancer via HER2 pathway inhibition, HER2 was stably knocked down in human HR^+^/HER2-low breast cancer cells. These cells were administered CDK4/6 inhibitor + endocrine therapy and triple combination treatment (neratinib, CDK4/6 inhibitor, and endocrine therapy). Rigorous assessment by the CCK-8 and colony formation assays confirmed that HER2 knockdown in both groups significantly inhibited the proliferation of HR^+^/HER2-low breast cancer cells (Fig. 6A,B). Further in vitro assays by cell flow cytometry and immunoblot conclusively confirmed substantial induction of G1 phase arrest, accompanied by reduced Cyclin D1 and CDK4 expression after neratinib intervention (Fig. 5G and Fig. 6C, D**)**. In addition, neratinib considerably reduced HER2 mRNA expression, according to the above results. Actinomycin D was employed to identify the regulatory effect of neratinib on HER2 mRNA stability, which helped elucidate the mechanism by which neratinib downregulates HER2 mRNA. The triple combination treatment group administered neratinib, CDK4/6 inhibitor, and endocrine therapy after actinomycin D intervention revealed significantly reduced HER2 mRNA stability at 5 and 10 h (Fig. 6E**)** compared with the CDK4/6 inhibitor + endocrine therapy group. Considering that miRNA plays a significant regulatory role in gene expression as a dynamic regulator of gene expression. The CDK4/6 inhibitor was examined in combination with the endocrine therapy and triple combination treatment groups, where microRNA sequencing was performed after neratinib, CDK4/6 inhibitor, and endocrine therapy, to assess whether miRNA could be altered with the addition of neratinib. The triple combination treatment group administered neratinib, CDK4/6 inhibitor, and endocrine therapy had significantly increased 39 miRNA amounts, according to expression profile analysis of sequencing data, compared with the CDK4/6 inhibitor + endocrine therapy group (Fig. 6F). Next, TargetScan (version 7.2, https://www.targetscan.org/vert_72/) was employed to predict target genes for the 39 upregulated miRNAs, and hsa-miR-23a-5p was found to highly bind the HER2’s 3’-UTR region. Simultaneously, microRNA sequencing data showed that compared with CDK4/6 inhibitor + endocrine therapy group, the triple combination treatment group administered neratinib, CDK4/6 inhibitor and endocrine therapy had significantly upregulated hsa-miR-23a-5p (Fig. 6G). Subsequent luciferase reporter gene assays demonstrated that hsa-miR-23a-5p binds substantially to HER2’s 3’-UTR region (Fig. 6H). After 5 and 10 h of actinomycin D intervention, hsa-miR-23a-5p mimic dramatically lowered HER2 mRNA stability (Fig. 6I) and downregulated HER2 and EGFR protein expression (Fig. 6J). Importantly, this intervention synergistically amplified the efficacy of CDK4/6 inhibitor combined with endocrine therapy in HR^+^/HER2-low breast cancer by suppressing the HER2 pathway.

### Optimization of neratinib dosing for enhanced CDK4/6 inhibitor combined with endocrine therapy efficacy

**Fig. 7:**
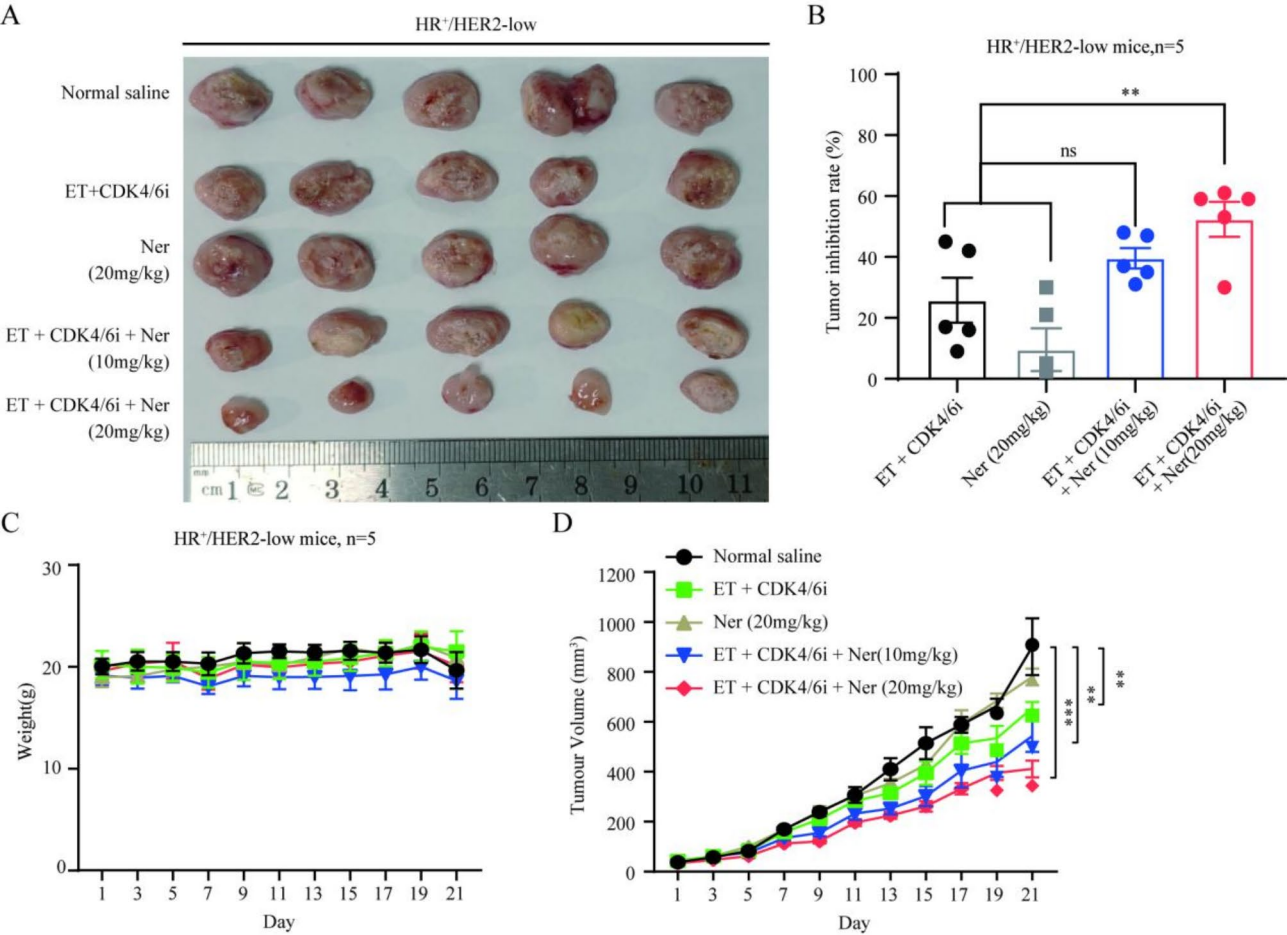
Optimization of neratinib dosing for enhanced CDK4/6 inhibitor combined with endocrine therapy efficacy. (**A**) Tumor photographs in animals treated with normal saline (control group), CDK4/6 inhibitor (palbociclib, 150 mg/kg, i.g.) + endocrine therapy (fulvestrant, 100 mg/kg, i.m.), medium-dose neratinib (20 mg/kg) alone, a triple therapy consisting of medium-dose neratinib group (20 mg/kg, i.g.), CDK4/6 inhibitor, and endocrine therapy, and a triple therapy consisting of low-dose neratinib (10 mg/kg, i.g.), CDK4/6 inhibitor, and endocrine therapy. (**C**) Weight changes in various groups of HR^+^/HER2-low tumor-bearing mice. (**B**) Tumor inhibition rates and (**D**) tumor growth curves of HR^+^/HER2-low tumor-bearing mice in various groups. For HER2-low tumor volume on day 21: normal saline vs. medium-dose ET + CDK4/6i + Ner, *P =* 0.0001; normal saline vs. low-dose ET + CDK4/6i + Ner, *P =* 0.0017; normal saline vs. ET + CDK4/6i, *P =* 0.003; ET + CDK4/6i vs. medium-dose ET + CDK4/6i + Ner, *P =* 0.0021. For HER2-low tumor inhibition rate on day 21: normal saline vs. medium-dose ET + CDK4/6i + Ner, *P =* 0.0217; normal saline vs. low-dose ET + CDK4/6i + Ner, *P =* 0.1271. ^**^P < 0.01; ^***^P < 0.001; *n* = 5. *ET* endocrine therapy, *CDK4/6i* CDK4/6 inhibitor, *Ner* neratinib.

Given the limited tolerance of the standard dose of neratinib (40 mg/kg) when combined with CDK4/6 inhibitor and endocrine therapy, we sought to optimize the dosing regimen for neratinib. Specifically, several groups were established, including a normal saline control group; a group administered CDK4/6 inhibitor combined with endocrine therapy; a triple therapy group administered a medium-dose of neratinib (20 mg/kg), CDK4/6 inhibitor, and endocrine therapy; a low-dose neratinib triple therapy group (10 mg/kg); and a separate group administered a medium-dose of neratinib (20 mg/kg) alone. Remarkably, mouse experiments unambiguously revealed that the medium dose of neratinib not only retained its potential to enhance the efficacy of CDK4/6 inhibitor combined with endocrine therapy in HR^+^/HER2-low breast cancer but also had good tolerability (*P* < 0.001). However, medium-dose neratinib alone had significantly compromised effectiveness (*P* < 0.01) (Fig. 7A-D). This insightful finding indicated that in patients with HR^+^/HER2-low breast cancer neratinib dosage might be optimized to be less intense than reported by studies examining HER2-positive cases. Significantly, even at lower doses, neratinib amplified the therapeutic potential of CDK4/6 inhibitor combined with endocrine therapy in HR^+^/HER2-low breast cancer.

## Discussion

This study applied meticulous experimentation involving HR^+^/HER2-low cell lines and animal models, highlighting the role of the HER2 pathway in the efficacy of CDK4/6 inhibitor combined with endocrine therapy for HR^+^/HER2-low breast cancer. This research further confirmed the decreased effectiveness of CDK4/6 inhibitor in combination with endocrine therapy in the HR^+^/HER2-low subgroup. Strikingly, this investigation also demonstrated that neratinib reversed the efficacy of CDK4/6 inhibitor and endocrine therapy by reducing HER2 mRNA stability in HR^+^/HER2-low breast cancer via the interaction of HER2’s 3’-UTR region with hsa-miR-23a-5p, and discovered that even when reducing neratinib dosage to the standard half dose (20 mg/kg), it remained highly effective and well-tolerated. Notably, the administered neratinib dose considerably deviated from current clinical standards, providing an innovative perspective on a potent, well-tolerated, and enhanced treatment strategy for HR^+^/HER2-low breast cancer, extending beyond CDK4/6 inhibitors combined with endocrine therapy.

Previous reports have demonstrated the variable responsiveness of HR^+^/HER2-low breast cancer to the combination of CDK4/6 inhibitors with endocrine therapy. Another study revealed a favorable outcome when utilizing an aromatase inhibitor combined with palbociclib within the HR^+^/HER2-low subtype. In contrast, discrepant data emanated from the Elizabeth Hospital of Hong Kong, where the median progression-free survival (mPFS) of fulvestrant combined with palbociclib in the HR^+^/HER2-low subtype had a notably decreased duration, accompanied by a corresponding reduction in efficacy^16,17^. Totally 11.7–14.8% of HR^+^/HER2-low cases had HER2-enrich phenotypes, according to a retrospective analysis of the MONALEESA study^20^. Consequently, the clinical data may be affected if the fraction of HER2 varies. In summary, we studied the effects of CDK4/6 inhibitor in combination with endocrine therapy in HR^+^/HER2-low and HR^+^/HER2-0 breast cancer cell subtypes both in vitro and in vivo.

Extensive in vitro and in vivo studies have unequivocally revealed the significant growth-inhibitory effects of CDK4/6 inhibitors combined with endocrine therapy in HR^+^/HER2-0 and HR^+^/HER2-low breast cancer cell subtypes. However, the efficacy of this regimen is remarkably hampered in HR^+^/HER2-low breast cancer compared with the HR^+^/HER2-0 subtype. Addressing this enigma, our innovative approach included knocking down HER2 in HR^+^/HER2-low breast cancer cells, resulting in a substantial enhancement of the inhibitory effects exerted by the combination of CDK4/6 inhibitor and endocrine therapy. Multiple mechanisms have been proposed to contribute to resistance to CDK4/6 inhibitors, including activation of the autophagy-lysosomal pathway, FAT1 deletion, FGFR1 activation, MDM2 dysregulation, mTOR activation, and regulated signaling pathways such as WEE1 and CDK7 overexpression^31–33^. These mechanisms may impair the efficacy of CDK4/6 inhibitors by disrupting the Cyclin D1-CDK4/6-Rb pathway, which regulates cell cycle progression^34–36^. This study examined the expression of Cyclin D1 and CDK4, key proteins involved in cell cycle regulation, revealing that knocking down HER2 in HR^+^/HER2-low breast cells cancer blocked the G1 phase and downregulated Cyclin D1 and CDK4. This suggests that HER2 is an important mediator in the transition of tumor cells from G1 to the S phase and represents an upstream pathway affecting the Cyclin D1-CDK4/6 pathway in HR^+^/HER2-low breast cancer^37–39^. The current findings support previous studies highlighting the significance of low HER2 expression in breast cancer^40,41^. Overall, this study indicates an imperative role for the HER2 pathway in determining the efficacy of CDK4/6 inhibitor combined with endocrine therapy for HR^+^/HER2-low breast cancer. Furthermore, even with attenuated HER2 expression in HR^+^/HER2-low breast cancer, the intricate interplay of HER2-associated pathways requires thorough consideration while investigating the mechanisms of resistance to CDK4/6 inhibitors in combination with endocrine therapy, thereby unveiling the complex landscape of treatment response^16,17^.

Pertinently, mTOR pathway activation has an adverse association with the efficacy of CDK4/6 inhibitors^32,42–44^. This intriguing finding prompted the TRINITI-1 trial, where a composite regimen encompassing a CDK4/6 inhibitor, an mTOR inhibitor, and an aromatase inhibitor was meticulously examined. Unfortunately, this approach yielded unsatisfactory outcomes, with a meager ORR of merely 8.4%^45,46^. In stark contrast, the noteworthy DESTINY-Breast 04 study unveiled an impressive ORR of 52.6% using the innovative ADC agent T-DXd in HR^+^/HER2-low breast cancer. The T-DXd treatment cohort exhibited remarkably enhanced PFS and OS versus the chemotherapy arm, thereby highlighting T-DXd’s profound potential as a pioneering targeted intervention for HER2-low breast cancer^47^. Furthermore, the efficacy spectrum is broadened by other ADC agents, corroborating their potency against HR^+^/HER2-low breast cancer^48,49^. Notably, this study centered on CDK4/6 inhibitor treatment. However, it left uncharted the unexplored field of combined therapy involving T-DXd, CDK4/6 inhibitors, and endocrine therapy. This study revealed that the triad combination of neratinib, CDK4/6 inhibitor, and endocrine therapy exerted superior suppression of HR^+^/HER2-low breast cancer growth. Nonetheless, an intriguing observation emerged as the standard neratinib dose (40 mg/kg) showed suboptimal tolerability in combination with CDK4/6 inhibitor and endocrine therapy. In a captivating twist, halving of neratinib dose (20 mg/kg, medium dose) not only retained its efficacy but also improved tolerability (*P* < 0.01). Intriguingly, further dose reduction to one-fourth (10 mg/kg, low dose) resulted in attenuated efficacy (*P* < 0.05). This dynamic interplay suggests that the required dosage of neratinib may be extraneous for HR^+^/HER2-low patients, particularly informed by HER2-positive breast cancer studies. These findings collectively lay a robust experimental foundation, substantiating the clinical viability of a novel regimen comprising medium-dose neratinib, CDK4/6 inhibitor combined with endocrine therapy, offering an important avenue for optimizing treatment in HR^+^/HER2-low breast cancer.

To determine the reason behind the efficacy difference between the two-drug regimen of CDK 4/6 inhibitor combined with endocrine therapy and the three-drug regimen comprising CDK 4/6 inhibitor, endocrine therapy combined with neratinib in HR^+^/HER2-low breast cancer, we also analyzed HER2 mRNA levels. We knocked down HER2 in HR^+^/HER2-low breast cancer cells and found no difference in the inhibitory effects of the three-agent regimen including CDK 4/6 inhibitor, endocrine therapy combined with neratinib on cell proliferation. These findings suggest that HER2 mRNA expression in HR^+^/HER2-low breast cancer correlates with the efficacy of CDK 4/6 inhibitors combined with endocrine therapy. Intriguingly, this finding aligns seamlessly with the retrospective MONALEESA study, where PAM50 analysis revealed that the clinical efficacy of CDK 4/6 inhibitors combined with endocrine therapy is not universally compromised in HR^+^/HER2-low breast cancers, and subsets such as luminal B, HER2-enriched, and basic-like phenotypes had notably lower efficacy compared with the luminal A subtype^7^. This nuanced understanding partially untangles the web of discrepant findings in clinical trials, potentially explained by varying levels of HER2 mRNA expression in the enrolled cohorts^16,17^.

Furthermore, this study revealed a notable reduction in HER2 mRNA stability in HR^+^/HER2-low breast cancer patients administered CDK 4/6 inhibitors, endocrine therapy, and neratinib three-agent regimens in comparison with CDK 4/6 inhibitors combined with endocrine therapy. This was achieved by HER2’s 3’-UTR region interacting with hsa-miR-23a-5p. This phenomenon supports the notion that the effect of the small molecule neratinib on augmenting treatment efficacy in HR^+^/HER2-low breast cancer transcends the confines of the conventional HER2 pathway. Intriguingly, such augmentation might extend beyond HER2 protein regulation, encapsulating transcriptional regulation and miRNA hsa-miR-23a-5p modulation, an unknown mechanism in small molecule neratinib studies. Contextually, parallel insights emerged from the realm of anti-HER2 monoclonal antibodies. De Haas et al. revealed substantial reductions in HER2 mRNA after treatment with trastuzumab and pertuzumab in stage II-III HER2-positive breast cancer patients^50^. However, further investigation is required to examine whether anti-HER2 macromolecular monoclonal antibodies also enhance the efficacy of CDK 4/6 inhibitors combined with endocrine therapy in HR^+^/HER2-low breast cancer.

This study had limitations. First, the effects of CDK4/6 inhibitors and endocrine therapy separately on the HER2 pathway in HR^+^/HER2-low breast cancer were not examined. Therefore, our data do not address whether the ultimate improvement in the efficacy of neratinib combined with CDK4/6 inhibitor rely on the effects of CDK4/6 inhibitor, endocrine therapy, or both. This study also lacked dose adjustments for palbociclib and fulvestrant, which prevents the determination of the optimal combination of the three drugs. Additionally, the cell lines used were identified as HR^+^/HER2-low according to public databases, and these limitations may affect the current findings and conclusions.

The current investigation demonstrated a significant relationship between hsa-miR-23a-5p expression in low HR^+^/HER2-low breast cancer and neratinib, affecting CDK4/6 inhibitor and endocrine therapy. However, a critical aspect remains untapped, i.e., the determination of the precise threshold of hsa-miR-23a-5p expression that substantively affects the efficacy of CDK 4/6 inhibitor combined with endocrine therapy. This uncharted field warrants further investigation to illuminate the future clinical translation prospects of the “medium-dose Ner + ET + CDK4/6i” triple combination therapy. We need to clarify this in future studies based on clinical samples. In addition, we advocate the systematic inclusion of HR^+^/HER2-low breast cancer patient-derived xenograft (PDX) and organoid models in future studies, which represents a promising avenue to unravel this intricate aspect. At the time, it is important to note that in vitro and mouse studies may lack direct clinical relevance in predicting the effectiveness or tolerability of triple combinations in clinical setting. Additionally, it should be acknowledged that T-DXd is currently established as the standard of care for patients with advanced, pretreated, HER2-low breast cancer based on current literature and clinical guidelines. Therefore, it is necessary to further validate the findings of this study in the clinic.

## Conclusions

This study newly found that the HER2 pathway is crucial in modulating the response to CDK4/6 inhibitors and endocrine therapy in HR^+^/HER2-low breast cancer. Neratinib can, through HER2’s 3’-UTR region binding hsa-miR-23a-5p and reducing HER2 mRNA stability, effectively downregulate Cyclin D1 and CDK4 by targeting the HER2 pathway, resulting in G1 arrest, and cancer cell proliferation, with enhanced efficacy of CDK4/6 inhibitors combined with endocrine therapy in HR^+^/HER2-low breast cancer. These findings provide significant insights into the development of effective and tolerable three-drug combination regimens applying “medium-dose Ner + ET + CDK4/6i” for clinical HR^+^/HER2-low breast cancer.

## Electronic supplementary material

Below is the link to the electronic supplementary material.

Supplementary Material 1

## Data Availability

The data sets used to support the findings of this study are available from the corresponding author upon request.

## References

[1] Giuliano, M. et al. Endocrine treatment versus chemotherapy in postmenopausal women with hormone receptor-positive, HER2-negative, metastatic breast cancer: a systematic review and network meta-analysis. *Lancet Oncol.* **20** (10), 1360–1369 (2019). 31494037 10.1016/S1470-2045(19)30420-6

[2] Ha, M. J. et al. Palbociclib plus endocrine therapy significantly enhances overall survival of HR+/HER2- metastatic breast cancer patients compared to endocrine therapy alone in the second-line setting: a large institutional study. *Int. J. Cancer*. **150** (12), 2025–2037 (2022). 35133007 10.1002/ijc.33959 PMC9018572

[3] Zhu, Z. et al. Comparative biomarker analysis of PALOMA-2/3 trials for palbociclib. *NPJ Precis. Oncol.* **6** (1), 56 (2022). 35974168 10.1038/s41698-022-00297-1 PMC9381541

[4] Hortobagyi, G. N. et al. Updated results from MONALEESA-2, a phase III trial of first-line ribociclib plus letrozole versus placebo plus letrozole in hormone receptor-positive, HER2-negative advanced breast cancer. *Ann. Oncol.* **29** (7), 1541–1547 (2018). 29718092 10.1093/annonc/mdy155

[5] Johnston, S. et al. MONARCH 3 final PFS: a randomized study of abemaciclib as initial therapy for advanced breast cancer. *NPJ Breast Cancer*. **5**, 5 (2019). 30675515 10.1038/s41523-018-0097-z PMC6336880

[6] Slamon, D. J. et al. Overall survival with ribociclib plus fulvestrant in advanced breast cancer. *N. Engl. J. Med.* **382** (6), 514–524 (2020). 31826360 10.1056/NEJMoa1911149

[7] Johnston, S. R. D. et al. Abemaciclib combined with endocrine therapy for the adjuvant treatment of HR+, HER2-, node-positive, high-risk, early breast cancer (monarchE). *J. Clin. Oncol.* **38** (34), 3987–3998 (2020). 32954927 10.1200/JCO.20.02514 PMC7768339

[8] Schettini, F. et al. Intrinsic subtypes and therapeutic decision-making in hormone receptor-positive/HER2-negative metastatic breast cancer with visceral crisis: a case report. *Front. Oncol.* **12**, 1009352 (2022). 36425558 10.3389/fonc.2022.1009352 PMC9679790

[9] Corona, S. P. & Generali, D. Abemaciclib: a CDK4/6 inhibitor for the treatment of HR+/HER2- advanced breast cancer. *Drug Des. Dev. Ther.* **12**, 321–330 (2018). 10.2147/DDDT.S137783 PMC5818877 29497278

[10] Alves, F. R. et al. Impact of human epidermal growth factor receptor 2 (HER2) low status in response to neoadjuvant chemotherapy in early breast cancer. *Cureus* **14** (2), e22330 (2022). 35371692 10.7759/cureus.22330 PMC8938239

[11] Wolff, A. C. et al. Human epidermal growth factor receptor 2 testing in breast cancer: American Society of Clinical Oncology/College of American Pathologists Clinical Practice Guideline Focused Update. *Arch. Pathol. Lab. Med.* **142** (11), 1364–1382 (2018). 29846104 10.5858/arpa.2018-0902-SA

[12] Fehrenbacher, L. et al. NSABP B-47/NRG oncology phase III randomized trial comparing adjuvant chemotherapy with or without Trastuzumab in high-risk invasive breast Cancer negative for HER2 by FISH and with IHC 1 + or 2. *J. Clin. Oncol.* **38** (5), 444–453 (2020). 31821109 10.1200/JCO.19.01455 PMC7007289

[13] Schettini, F. et al. Clinical, pathological, and PAM50 gene expression features of HER2-low breast cancer. *NPJ Breast Cancer*. **7** (1), 1 (2021). 33397968 10.1038/s41523-020-00208-2 PMC7782714

[14] Shui, R. et al. Hormone receptor and Human Epidermal Growth Factor Receptor 2 detection in invasive breast carcinoma: a retrospective study of 12,467 patients from 19 Chinese Representative Clinical centers. *Clin. Breast Cancer*. **20** (1), e65–e74 (2020). 31669267 10.1016/j.clbc.2019.07.013

[15] Rossi, V. et al. Moderate immunohistochemical expression of HER-2 (2+) without HER-2 gene amplification is a negative prognostic factor in early breast cancer. *Oncologist* **17** (11), 1418–1425 (2012). 22951668 10.1634/theoncologist.2012-0194 PMC3500362

[16] Bao, K. K. H., Sutanto, L., Tse, S. S. W., Man Cheung, K. & Chan, J. C. H. The Association of ERBB2-Low expression with the efficacy of cyclin-dependent kinase 4/6 inhibitor in hormone Receptor-Positive, ERBB2-Negative metastatic breast Cancer. *JAMA Netw. Open.* **4** (11), e2133132 (2021). 34739066 10.1001/jamanetworkopen.2021.33132 PMC8571658

[17] Carlino, F. et al. HER2-Low Status does not affect survival outcomes of patients with metastatic breast Cancer (MBC) Undergoing First-Line treatment with endocrine therapy plus Palbociclib: results of a Multicenter, Retrospective Cohort Study. *Cancers (Basel)* **14**(20) (2022). 10.3390/cancers14204981 PMC9599946 36291765

[18] Xia, L. Y., Cao, X. C. & Yu, Y. Survival outcomes in HER2-low versus HER2-zero breast cancer after neoadjuvant chemotherapy: a meta-analysis. *World J. Surg. Oncol.* **22** (1), 106 (2024). 38643188 10.1186/s12957-024-03382-w PMC11031865

[19] Bidard, F. C. et al. Switch to fulvestrant and palbociclib versus no switch in advanced breast cancer with rising ESR1 mutation during aromatase inhibitor and palbociclib therapy (PADA-1): a randomised, open-label, multicentre, phase 3 trial. *Lancet Oncol.* **23** (11), 1367–1377 (2022). 36183733 10.1016/S1470-2045(22)00555-1

[20] Prat, A. et al. Correlative biomarker analysis of intrinsic subtypes and efficacy across the MONALEESA Phase III studies. *J. Clin. Oncol.* **39** (13), 1458–1467 (2021). 33769862 10.1200/JCO.20.02977 PMC8196091

[21] Maddox, A. L. et al. Molecular Assessment of HER2 to Identify Signatures Associated with Therapy Response in HER2-Positive breast Cancer. *Cancers (Basel)* **14**(11) (2022). 10.3390/cancers14112795 PMC9179327 35681773

[22] Carey, L. A. et al. Molecular heterogeneity and response to Neoadjuvant Human Epidermal Growth Factor Receptor 2 targeting in CALGB 40601, a Randomized Phase III Trial of Paclitaxel Plus Trastuzumab with or without Lapatinib. *J. Clin. Oncol.* **34** (6), 542–549 (2016). 26527775 10.1200/JCO.2015.62.1268 PMC4980567

[23] Ganz, P. A. et al. NRG Oncology/NSABP B-47 menstrual history study: impact of adjuvant chemotherapy with and without trastuzumab. *NPJ Breast Cancer*. **7** (1), 55 (2021). 34016989 10.1038/s41523-021-00264-2 PMC8137688

[24] Schneeweiss, A. et al. Phase ib study evaluating safety and clinical activity of the anti-HER3 antibody lumretuzumab combined with the anti-HER2 antibody pertuzumab and paclitaxel in HER3-positive, HER2-low metastatic breast cancer. *Investig. New Drugs* **36** (5), 848–859 (2018). 29349598 10.1007/s10637-018-0562-4 PMC6153514

[25] Jhaveri, K. L. et al. Neratinib plus fulvestrant plus trastzuzumab (N + F + T) for hormone receptor-positive (HR+), HER2-negative, HER2-mutant metastatic breast cancer (MBC): outcomes and biomarker analysis from the SUMMIT trial. *J. Clin. Oncol.* **40** (16_suppl), 1028–1028 (2022).

[26] Collins, D. M. et al. Effects of HER Family-targeting tyrosine kinase inhibitors on antibody-dependent cell-mediated cytotoxicity in HER2-expressing breast Cancer. *Clin. Cancer Res.* **27** (3), 807–818 (2021). 33122343 10.1158/1078-0432.CCR-20-2007 PMC7854527

[27] Yu, Y., Huang, K., Lin, Y., Zhang, J. & Song, C. Tyrosine kinase inhibitors in HER2-positive breast cancer brain metastases: a systematic review and meta-analysis. *Cancer Med.* **12** (14), 15090–15100 (2023). 37255389 10.1002/cam4.6180 PMC10417165

[28] Lin, N. U. et al. Tucatinib vs Placebo, both in Combination with Trastuzumab and Capecitabine, for previously treated ERBB2 (HER2)-Positive metastatic breast Cancer in patients with brain metastases: updated exploratory analysis of the HER2CLIMB Randomized Clinical Trial. *JAMA Oncol.* **9** (2), 197–205 (2023). 36454580 10.1001/jamaoncol.2022.5610 PMC9716438

[29] Yan, M. et al. Pyrotinib plus capecitabine for patients with human epidermal growth factor receptor 2-positive breast cancer and brain metastases (PERMEATE): a multicentre, single-arm, two-cohort, phase 2 trial. *Lancet Oncol.* **23** (3), 353–361 (2022). 35085506 10.1016/S1470-2045(21)00716-6

[30] Greer, Y. E. et al. Mitochondrial matrix protease ClpP agonists inhibit Cancer stem cell function in breast Cancer cells by disrupting mitochondrial homeostasis. *Cancer Res. Commun.* **2** (10), 1144–1161 (2022). 36388465 10.1158/2767-9764.CRC-22-0142 PMC9645232

[31] Vijayaraghavan, S. et al. CDK4/6 and autophagy inhibitors synergistically induce senescence in rb positive cytoplasmic cyclin E negative cancers. *Nat. Commun.* **8**, 15916 (2017). 28653662 10.1038/ncomms15916 PMC5490269

[32] Scheidemann, E. R. & Shajahan-Haq, A. N. Resistance to CDK4/6 inhibitors in Estrogen receptor-positive breast Cancer. *Int. J. Mol. Sci.* **22**(22) (2021). 10.3390/ijms222212292 PMC8625090 34830174

[33] Pancholi, S. et al. Tumour kinome re-wiring governs resistance to palbociclib in oestrogen receptor positive breast cancers, highlighting new therapeutic modalities. *Oncogene* **39** (25), 4781–4797 (2020). 32307447 10.1038/s41388-020-1284-6 PMC7299844

[34] Herrera-Abreu, M. T. et al. Early adaptation and acquired resistance to CDK4/6 inhibition in Estrogen receptor-positive breast cancer. *Cancer Res.* **76** (8), 2301–2313 (2016). 27020857 10.1158/0008-5472.CAN-15-0728 PMC5426059

[35] Stanciu, I. M. et al. Mechanisms of resistance to CDK4/6 inhibitors and predictive biomarkers of response in HR+/HER2-Metastatic breast Cancer-A review of the literature. *Diagnostics (Basel)* **13**(5) (2023). 10.3390/diagnostics13050987 PMC10000620 36900131

[36] Pandey, K. et al. Molecular mechanisms of resistance to CDK4/6 inhibitors in breast cancer: a review. *Int. J. Cancer* **145** (5), 1179–1188 (2019). 30478914 10.1002/ijc.32020 PMC6767051

[37] O’Leary, B., Finn, R. S. & Turner, N. C. Treating cancer with selective CDK4/6 inhibitors. *Nat. Rev. Clin. Oncol.* **13** (7), 417–430 (2016). 27030077 10.1038/nrclinonc.2016.26

[38] Alves, C. L. et al. High CDK6 protects cells from fulvestrant-mediated apoptosis and is a predictor of resistance to Fulvestrant in Estrogen receptor-positive metastatic breast Cancer. *Clin. Cancer Res.* **22** (22), 5514–5526 (2016). 27252418 10.1158/1078-0432.CCR-15-1984

[39] VanArsdale, T., Boshoff, C., Arndt, K. T. & Abraham, R. T. Molecular pathways: targeting the cyclin D-CDK4/6 Axis for Cancer Treatment. *Clin. Cancer Res.* **21** (13), 2905–2910 (2015). 25941111 10.1158/1078-0432.CCR-14-0816

[40] Eiger, D., Agostinetto, E., Saude-Conde, R. & de Azambuja, E. The Exciting New Field of HER2-low breast cancer treatment. *Cancers (Basel)* **13**(5) (2021). 10.3390/cancers13051015 PMC7957750 33804398

[41] Gao, A. et al. LEM4 confers tamoxifen resistance to breast cancer cells by activating cyclin D-CDK4/6-Rb and ERalpha pathway. *Nat. Commun.* **9** (1), 4180 (2018). 30301939 10.1038/s41467-018-06309-8 PMC6177406

[42] Cortes, J. et al. The next era of treatment for hormone receptor-positive, HER2-negative advanced breast cancer: Triplet combination-based endocrine therapies. *Cancer Treat. Rev.* **61**, 53–60 (2017). 29100169 10.1016/j.ctrv.2017.09.011

[43] Cai, Z. et al. Overexpressed cyclin D1 and CDK4 proteins are responsible for the resistance to CDK4/6 inhibitor in breast cancer that can be reversed by PI3K/mTOR inhibitors. *Sci. China Life Sci.* **66** (1), 94–109 (2023). 35982377 10.1007/s11427-021-2140-8

[44] O’Brien, N. A. et al. Targeting activated PI3K/mTOR signaling overcomes acquired resistance to CDK4/6-based therapies in preclinical models of hormone receptor-positive breast cancer. *Breast Cancer Res.* **22** (1), 89 (2020). 32795346 10.1186/s13058-020-01320-8 PMC7427086

[45] Bardia, A. et al. Phase ib Dose-escalation/Expansion trial of Ribociclib in Combination with Everolimus and Exemestane in Postmenopausal Women with HR(+), HER2(-) advanced breast Cancer. *Clin. Cancer Res.* **26** (24), 6417–6428 (2020). 32998962 10.1158/1078-0432.CCR-20-1068 PMC8489181

[46] Bardia, A. et al. Phase I/II trial of Exemestane, Ribociclib, and Everolimus in Women with HR+/HER2(-) advanced breast Cancer after progression on CDK4/6 inhibitors (TRINITI-1). *Clin. Cancer Res.* **27** (15), 4177–4185 (2021). 33722897 10.1158/1078-0432.CCR-20-2114 PMC8487593

[47] Modi, S. et al. Trastuzumab Deruxtecan in previously treated HER2-Positive breast Cancer. *N. Engl. J. Med.* **382** (7), 610–621 (2020). 31825192 10.1056/NEJMoa1914510 PMC7458671

[48] Wang, J. Y. et al. RC48-ADC, a HER2-targeting antibody-drug conjugate, in patients with HER2-positive and HER2-low expressing advanced or metastatic breast cancer: a pooled analysis of two studies. *J. Clin. Oncol.* ;**39**(15) (2021).

[49] Banerji, U. et al. Trastuzumab duocarmazine in locally advanced and metastatic solid tumours and HER2-expressing breast cancer: a phase 1 dose-escalation and dose-expansion study. *Lancet Oncol.* **20** (8), 1124–1135 (2019). 31257177 10.1016/S1470-2045(19)30328-6

[50] de Haas, S. L. et al. Tumor biomarkers and efficacy in patients treated with trastuzumab emtansine + pertuzumab versus standard of care in HER2-positive early breast cancer: an open-label, phase III study (KRISTINE). *Breast Cancer Res.* **25** (1), 2 (2023). 36631725 10.1186/s13058-022-01587-z PMC9832665

