# Supplementary material for: Neratinib enhances the efficacy of CDK4/6 inhibitor plus endocrine therapy in HR+/HER2-low breast cancer cell line ZR-75-1 via hsa-miR-23a-5p
Source: Sci Rep. 2024 Dec 28;14:31062. doi: 10.1038/s41598-024-82137-9 (PMC11680982; doi:10.1038/s41598-024-82137-9)

Western blot original images

Fig.3A HER2

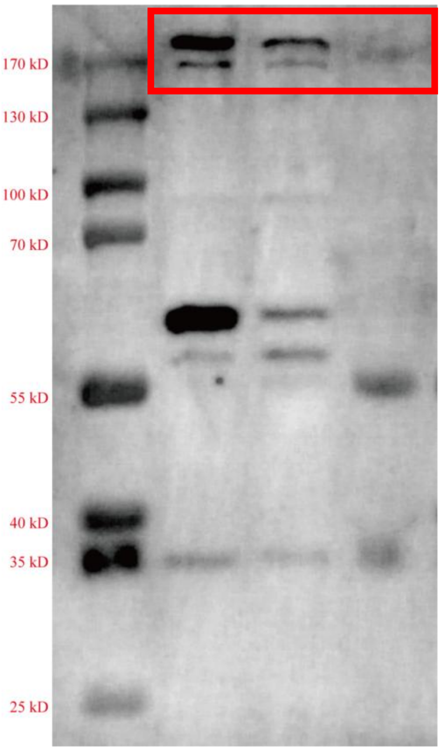

Fig.3A GAPDH

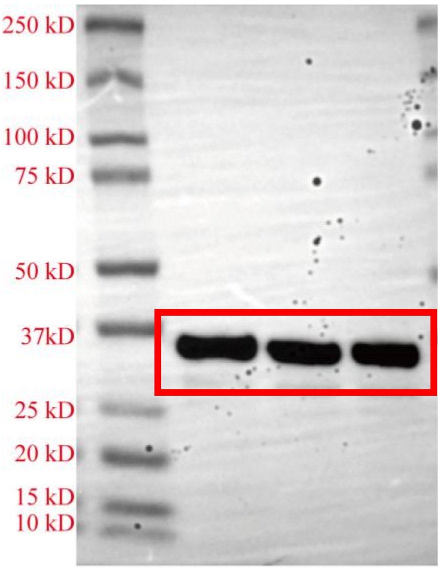

Fig.3G Cyclin D1

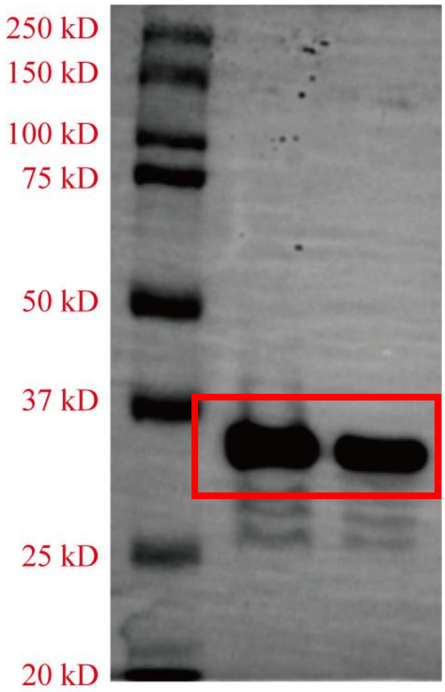

Fig.3G CDK4

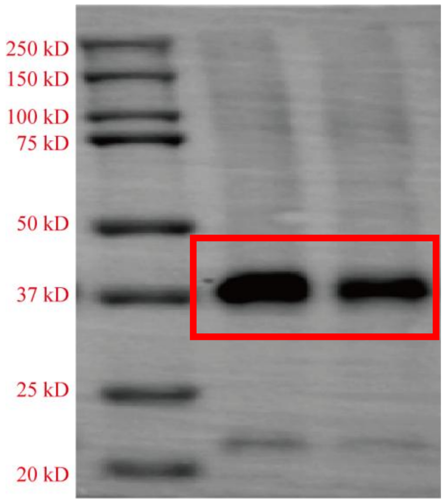

Fig.3G EGFR

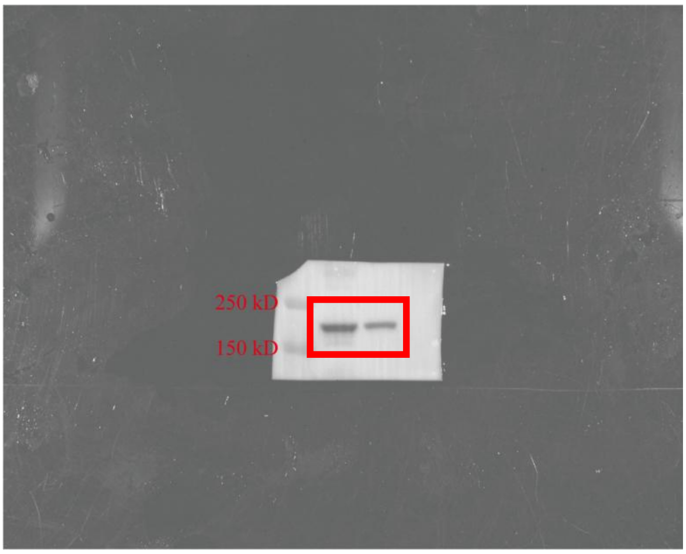

Fig.3G GAPDH

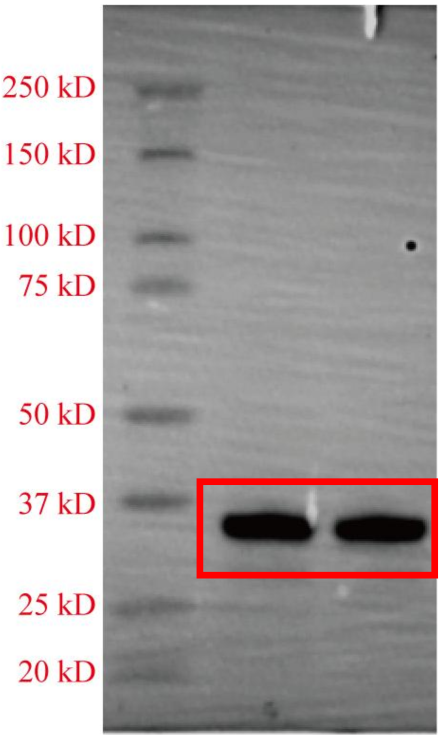

Fig.4D Cyclin D1

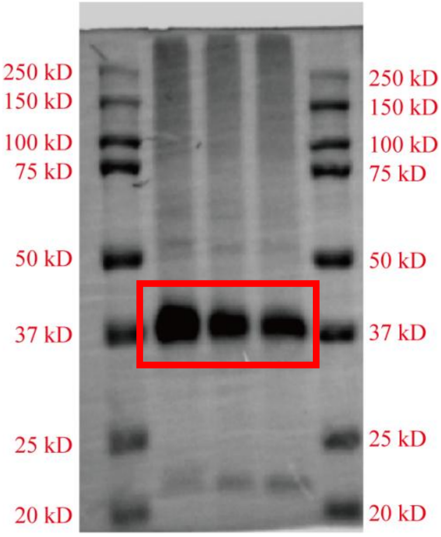

Fig.4D CDK4

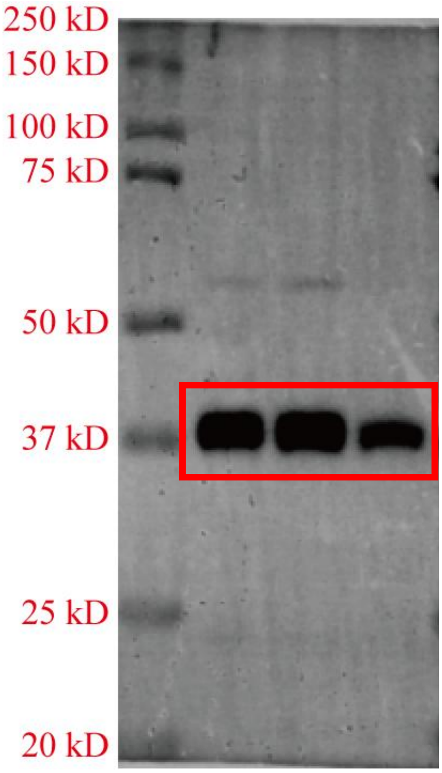

Fig.4D HER2

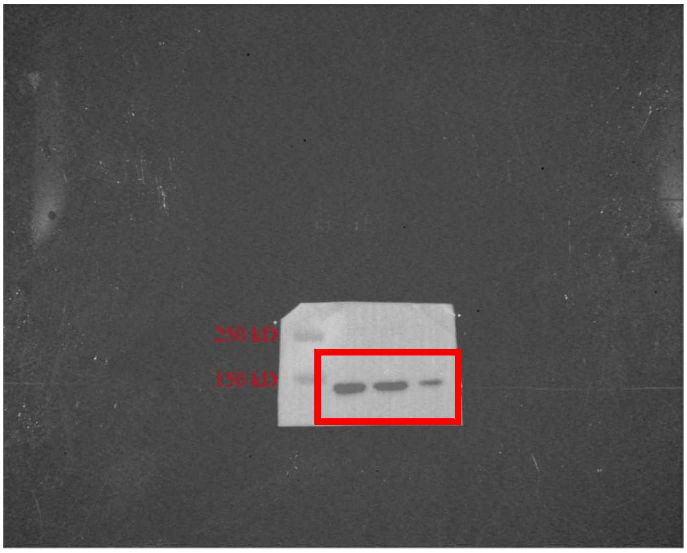

Fig.4D EGFR

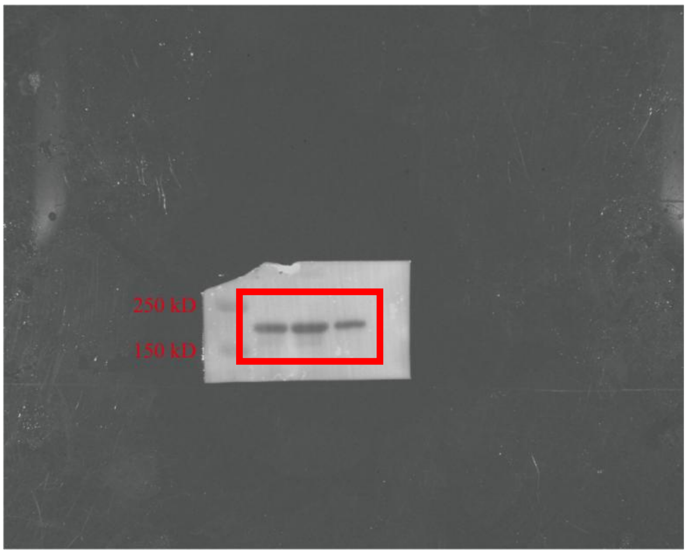

Fig.4D GAPDH

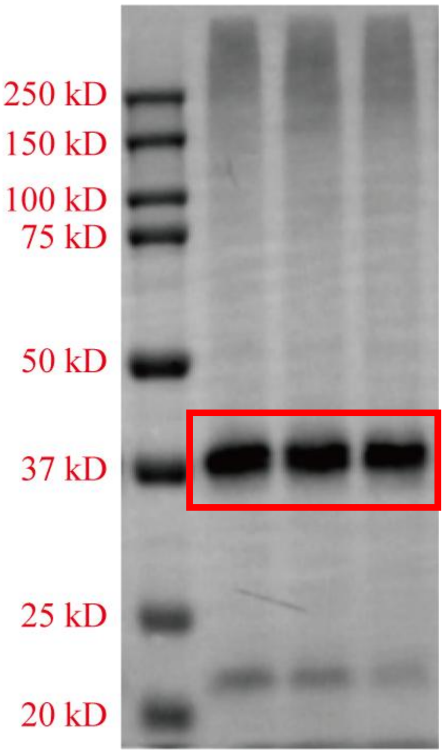

Fig.5G Cyclin D1

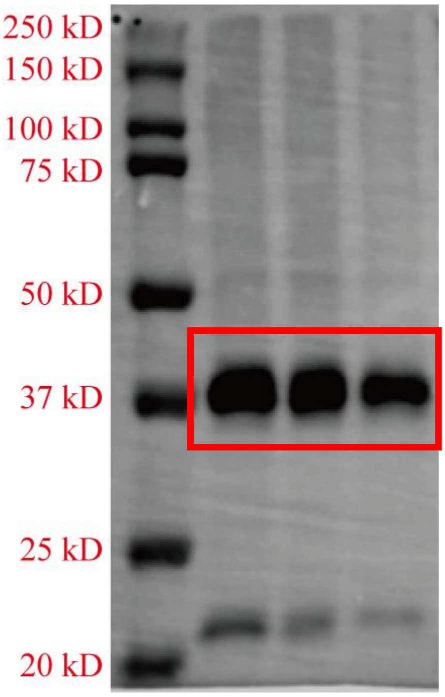

Fig.5G CDK4

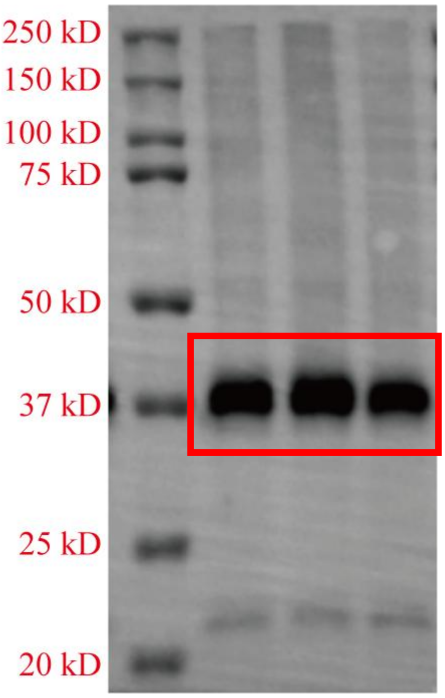

Fig.5G EGFR

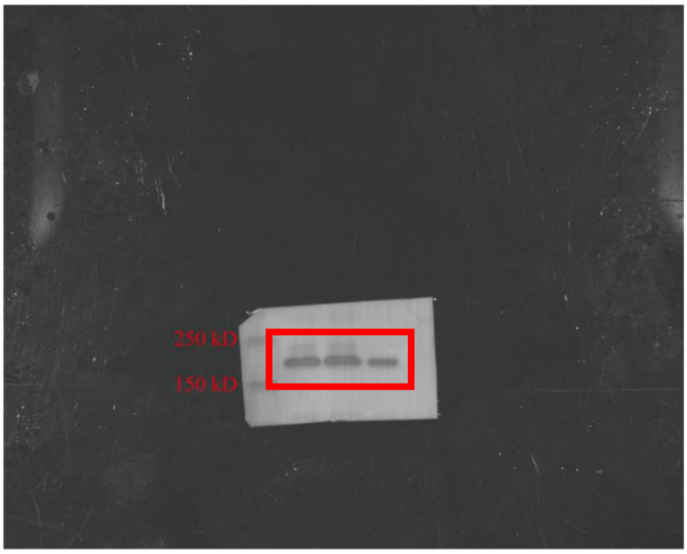

Fig.5G GAPDH

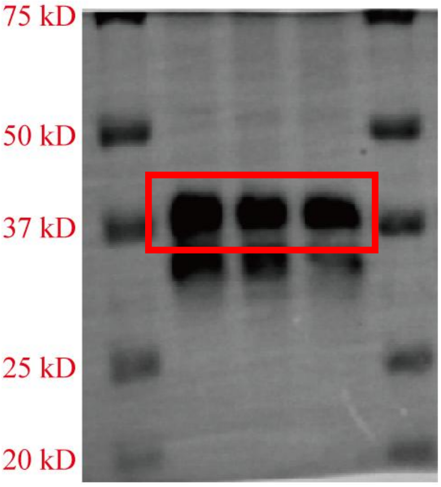

Fig.6D Cyclin D1

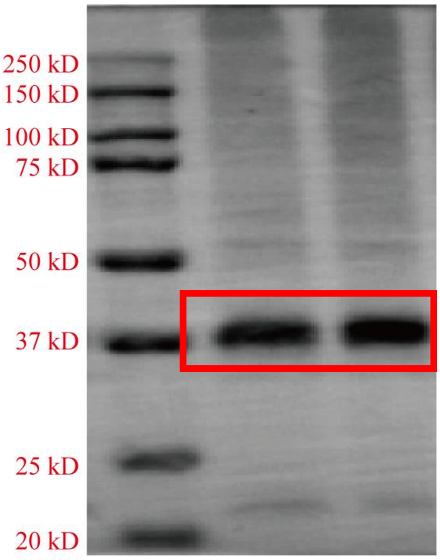

Fig.6D CDK4

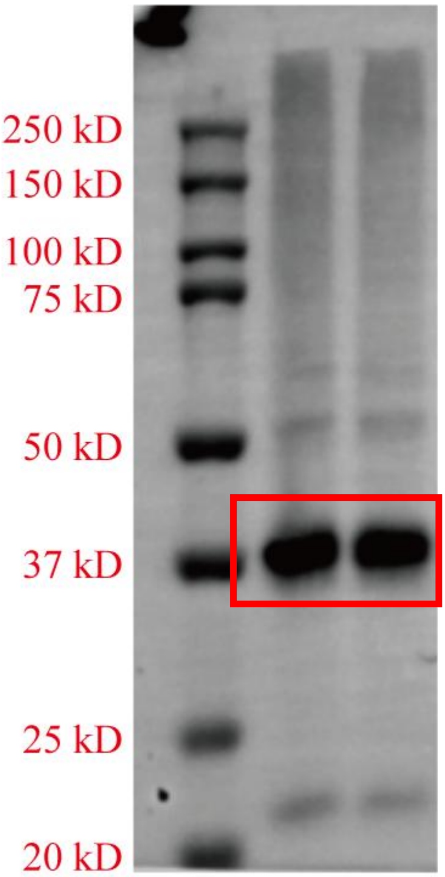

Fig.6D EGFR

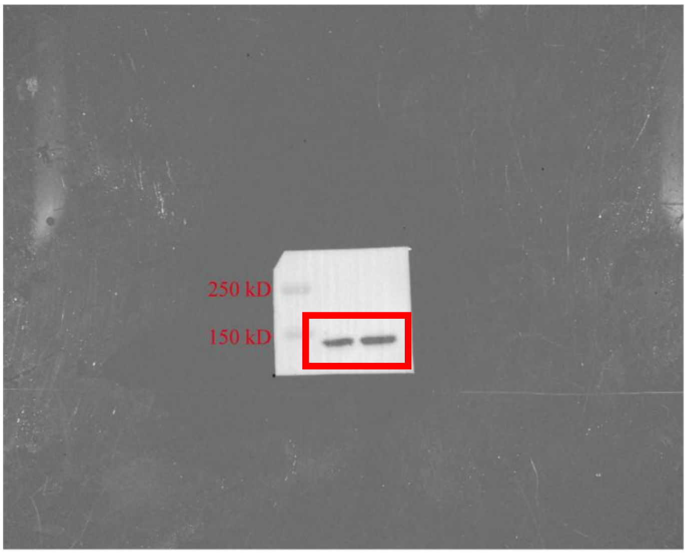

Fig.6D GAPDH

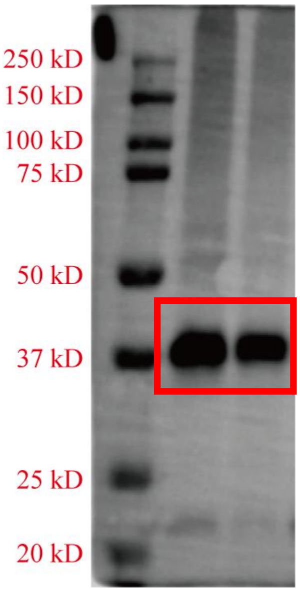

Fig.6J GAPDH/HER2

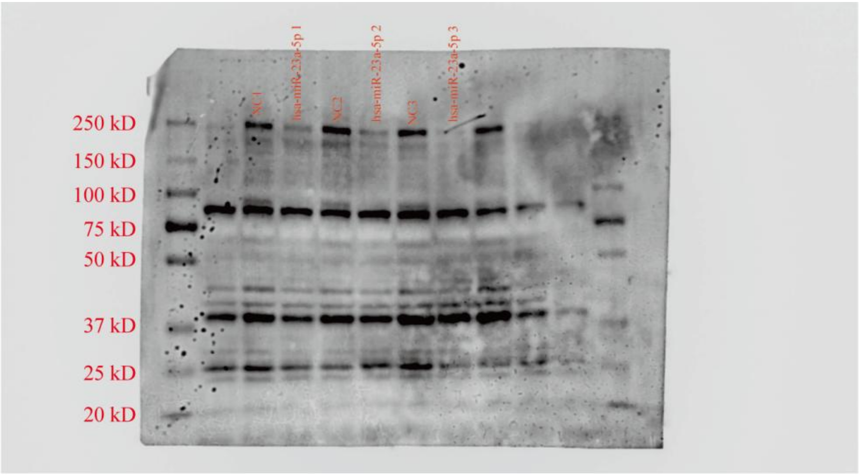

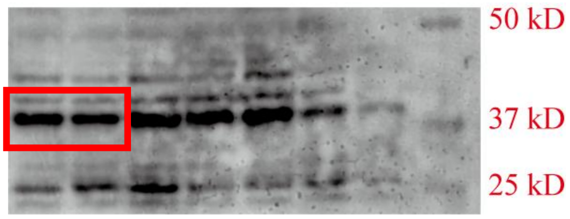

GAPDH

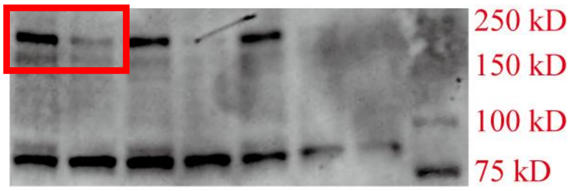

HER2

Fig.6J EGFR

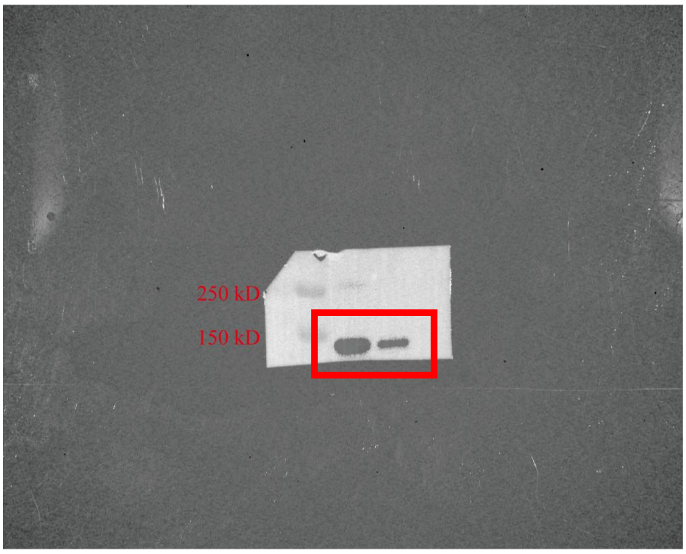

Supplement: Supplementary file 1 — Supplementary Material 1 [file 41598_2024_82137_MOESM1_ESM.pdf]
